# A novel extraction method enhanced the osteogenic and anti-osteoporosis effect of tea extract without any hepatotoxicity in ovariectomized rats

**DOI:** 10.3389/fendo.2022.951800

**Published:** 2022-08-08

**Authors:** Chirag Kulkarni, Shivani Sharma, Prateek Singh Bora, Saurabh Verma, Swati Rajput, Konica Porwal, Srikanta Kumar Rath, Jiaur Rahaman Gayen, Upendra Sharma, Naibedya Chattopadhyay

**Affiliations:** ^1^ Division of Endocrinology and Centre for Research in Anabolic Skeletal Targets in Health and Illness (ASTHI), CSIR-Central Drug Research Institute, Lucknow, India; ^2^ Academy of Scientific and Innovative Research (AcSIR), Ghaziabad, India; ^3^ Division of Chemical Technology, CSIR-Institute of Himalayan Bioresource Technology, Palampur, India; ^4^ Division of Pharmaceutics & Pharmacokinetics, CSIR-Central Drug Research Institute, Lucknow, India; ^5^ Division of Toxicology and Experimental Medicine, CSIR-Central Drug Research Institute, Lucknow, India

**Keywords:** acid hydrolysis, post-menopausal osteoporosis, hepatotoxicity, micro-computed tomography, histomorphometry, pharmacokinetics, preclinical toxicity

## Abstract

Tea (*Camellia sinensis*) has several reported health benefits, including that on bone health attributed to catechins of which the most abundant is epigallocatechin-3-gallate (EGCG). However, several preclinical and clinical studies raise safety concerns about EGCG in tea extract causing acute liver failure. Tea also contains kaempferol, albeit scanty, and it has hepatoprotective and osteogenic effects. Here, we utilized a novel extraction procedure of acid hydrolysis to enhance the osteogenic effect of tea extract while reducing its hepatotoxicity. The resultant extract (USKECSE) has a ~40-fold increase in kaempferol and a 2.5-fold reduction in EGCG content compared with the hydroethanolic extract (USCSE). In a female Sprague Dawley (SD) rat femur osteotomy model, USKECSE (100 mg/kg) but not USCSE promoted bone regeneration. In a rat postmenopausal osteoporosis model induced by bilateral ovariectomy (OVX), USKECSE through an osteogenic mechanism maintained bone mass, strength, and microarchitecture to the levels of ovary-intact rats with no hepatotoxic effect. After a single oral dose (100 mg/kg) of USKECSE to adult rats, kaempferol was detectable for 48 hours, suggesting its significant absorption and distribution in plasma. Peak kaempferol concentration in plasma (C_max_) was 483 ng/ml (2 μM), and at this concentration, kaempferol induces osteoblast differentiation. USKECSE had no genotoxicity, and its safety index assessed by preclinical toxicity studies, including safety pharmacology, was >20-fold. Taken together, we report a novel extraction process that enhanced the osteogenicity and concomitantly reduced hepatotoxicity of tea extract with significant kaempferol bioavailability and a favorable systemic safety profile. Based on these data, we propose assessing the USKECSE effect for postmenopausal osteoporosis treatment.

## 1 Introduction

Estrogen deficiency at menopause causes accelerated bone loss due to increased formation and function of the bone resorbing osteoclasts and increased apoptosis and diminished function of bone-forming osteoblasts (1, 2). Hormone replacement therapy (HRT) provides an effective preventive and therapeutic option for postmenopausal osteoporosis (PMO). However, in light of findings of safety issues among HRT users, including increased incidences of breast cancer, heart disease, stroke, and formation of clots, led to its discontinuation in postmenopausal women (3).

Currently, bisphosphonates are the first line of anti-osteoporosis therapy for PMO. However, gastrointestinal side effects and resultant poor compliance have led to an active search for a better and safer alternative to clinical management of PMO (4). Phytoestrogens represent a potential alternative to anti-osteoporosis drugs as these are structurally similar to the primary female sex hormone, 17β-estradiol (E2) (5). Isoflavonoids, including genistein and daidzein, have a binding affinity with E2 receptors (ERs) and are abundantly present in soybean. The effect of soy isoflavonoid extract on bone mineral density (BMD) in postmenopausal women is equivocal. A meta-analysis study by Taku et al. showed that soy isoflavone treatment results in BMD increase in the lumbar spine but not in femur neck, total hip, and trochanter (6), but in a different study, Liu et al. find no positive effect of soy isoflavone in spine BMD (7). Habitual tea drinking has shown some beneficial effects on bone; however, study designs are either cross-sectional or retrospective and yielded inconsistent conclusions (8, 9). Given the purported health benefits of habitual tea drinking, in particular, green tea, a plethora of green tea preparations as supplements are commercially available. The beneficial effects of green tea are attributed to catechins of which epigallocatechin gallate (EGCG) has been mostly implicated as both beneficial and harmful (10). The most publicized harmful effect of green tea extract (GTE) is hepatotoxicity reported from studies that administer repeated oral boluses of GTE (11).

Flavonols, particularly quercetin and kaempferol, are among the most widely distributed flavonoids in foods with diverse reported health benefits, including bone health (5). We have shown that kaempferol is more potent than quercetin for inhibiting osteoclastogenesis (12) and has an osteogenic effect in E2-deficient rats (13). Moreover, several preclinical studies reported the hepatoprotective effect of kaempferol in preclinical models of liver disease (14–16). Although catechins are the major green tea polyphenols, kaempferol is also present, albeit to a lesser extent (17). Therefore, substituting the hepatotoxic compound EGCG with the osteogenic compound kaempferol appears to be a novel approach to harness the beneficial bone effects of tea extract with a concomitant reduction of hepatotoxicity. Accordingly, we set out to prepare a tea extract enriched with kaempferol but diminished in EGCG content.

To this aim, we first prepared a polyphenol-rich extract (USCSE) from the leaves of *C. sinensis* and then subjected it to acid hydrolysis and obtained a kaempferol-enriched extract (USKECSE). We then tested the bone-regenerative effect of both extracts in a rat femur osteotomy model, which revealed that USKECSE was more potent than USCSE. In subsequent studies, we tested the skeletal effects of USKECSE in ovariectomized (OVX) rats through the assessments of bone mass, microarchitecture, bone strength, and bone formation and also checked their liver function. We also studied the oral bioavailability of kaempferol upon USKECSE administration. Finally, we assessed the preclinical safety and toxicity of USKECSE.

## 2 Material and methods

### 2.1 Chemicals and reagents

Cell culture medium, collagenase, and all chemicals were purchased from Sigma-Aldrich (St. Louis Missouri, USA). Standards of EGCG (**1**) and kaempferol (**2**), were procured from Sigma-Aldrich (St. Louis Missouri, USA). The purities of all standards were more than 98% as determined by UPLC analysis. Amberlyst A-21, the free base resin, was procured from Sigma-Aldrich (St. Louis Missouri, USA). Hydrochloric acid was purchased from Central Drug House. Formic acid was purchased from S. D. Fine Chemicals Ltd. (Mumbai, India), and solvents from J. T. Baker (Mallinckrodt Baker Inc., St. Louis, Missouri, USA) and S. D. Fine Chemicals Ltd. (Mumbai, India). Cell culture supplements, viz. FBS and diaspase, were purchased from Invitrogen (Carlsbad, California, USA). Gum acacia was purchased from Santa Cruz Biotechnology, Inc. (Dallas, Texas, United States).

### 2.2 Plant material

The leaves of *C. sinensis* were collected from the Banuri tea farm, CSIR-IHBT (36°N and 78.18°E, 1200 m above mean sea level) and were authenticated by a taxonomy expert at the Environment Technology division of CSIR-IHBT, Palampur, Himachal Pradesh, India.

### 2.3 Extraction

**Figure 1:**
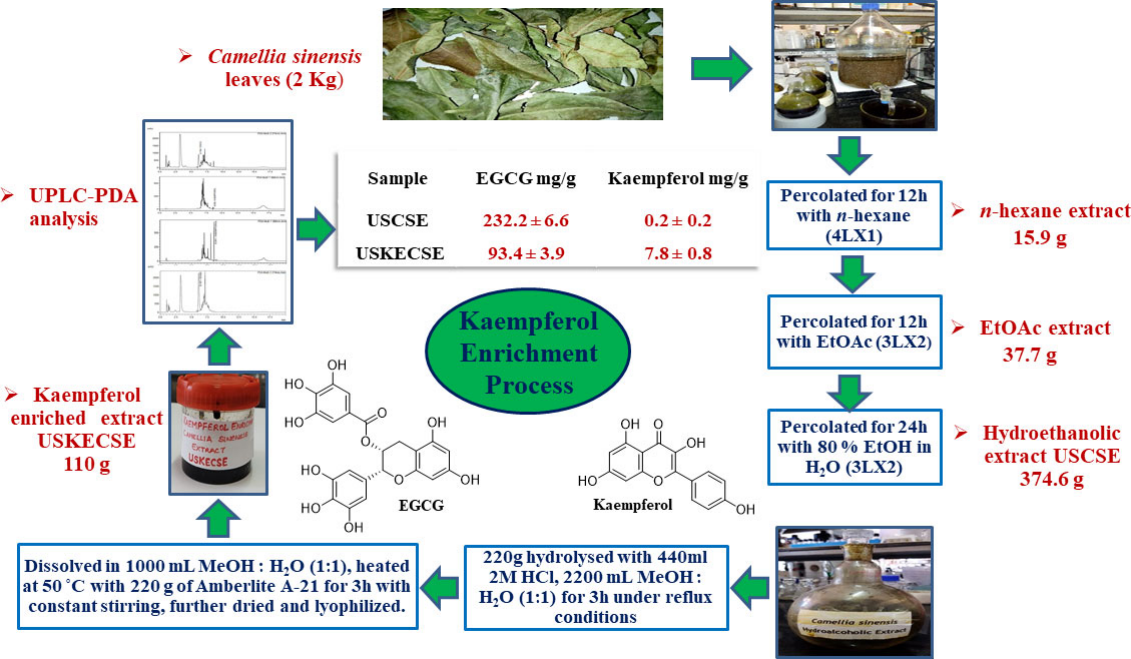
Schematic diagram showing the process of preparation of kaempferol-enriched extract (USKECSE) from *C. sinensis*.

Air-dried leaves (2 kg) of *C. sinensis* were powdered and sequentially percolated with *n-*hexane (4L x 1) for 12 hours with ethyl acetate (3L x 2) for 12 hours, and 80% ethanol in water (3L x 2) for 24 hours at room temperature. The percolates were combined and evaporated under reduced pressure at 50°C to give *n-*hexane (15.9 g), ethyl acetate (37.7 g), and hydroethanolic (374.6 g) extract (**Figure 1**).

### 2.4 Acid hydrolysis

*C. sinensis* hydroethanolic extract (220 g) was hydrolyzed with 2 M HCl (440 ml) in MeOH:H_2_O (1:1, 2200 ml) for three hours under reflux conditions. The resultant extract was dried at 50°C under reduced pressure. *C. sinensis* hydrolyzed extract (acidified) obtained from the hydrolysis experiment was again dissolved in MeOH:H_2_O (1:1, 1000 ml) and treated with 220 g of Amberlyst A-21 free base resin at 50°C for three hours with constant stirring. Amberlite A-21 resin was separated from the mixture by decantation and filtration. The solution was then dried at 50°C under reduced pressure and further lyophilized to obtain 110 g of kaempferol-enriched *C. sinensis* extract. The entire study was conducted using one batch of USCSE and USKECSE.

### 2.5 Sample preparation

Next, 20.8 mg of dried and powdered *C. sinensis* hydroethanolic extract (USCSE) and 16.6 mg of kaempferol-enriched *C. sinensis extract* (USKECSE) were accurately weighed and sonicated with LC grade methanol (1 ml) for 10 minutes. The solution was filtered with a 0.22-μm syringe filter, and samples were stored at 4°C before UPLC analysis.

### 2.6 Standard solutions

The reference standards, *i.e.*, EGCG and kaempferol, were accurately weighed and dissolved in methanol to prepare stock solutions. The concentration of stock solutions for each compound was 1.0 mg/mL. The stock solution was then serially diluted with methanol to give different concentrations in the range of 2.5–320 μg/mL for EGCG and 0.098–31.25 μg/ml for kaempferol. The prepared dilutions were stored at 4°C before analysis.

### 2.7 UPLC conditions

The quantitative analysis of samples was performed on the Shimadzu Nexera X-2 LC-30AD UPLC system coupled with the Shimadzu SPD-M30A PDA detector. The separation of analytes was carried out on the BEH-C18 column (2.1 mm × 100 mm, 1.7 μm) with a column temperature of 27°C. The eluents consisted of water (0.01% formic acid) as solvent A and acetonitrile as solvent B at a flow rate of 0.240 mL/min with a linear gradient programmed for 20 minutes as, 0.10–0.30 minutes, 10% B; 0.30–3.0 minutes, 10%–50% B; 3.0–10.50 minutes, 50%–55% B; 10.50–11.0 minutes, 55%–90% B; 11.0–12.0 minutes, 90%–90% B; 12.0–13.0 minutes, 90%–10% B; 13.0–20.0 minutes, 10% B. The injection volume was 2 μL for extracts and standard solutions and 274 nm and 366 nm were chosen as the target wavelength (λ max) for the detection of the two reference standards.

### 2.8 UPLC method validation

The UPLC method was validated for linearity, the limit of detection (LOD), limit of quantitation (LOQ), precision, and accuracy by ICH guidelines [1]. For the development of the calibration curve, eight different concentrations of mixed solutions of two reference standards were prepared, and the graph was plotted based on areas of standard *vs.* concentration of each analyte. To determine the LOD and LOQ, the lowest concentration of mixed standard solution was further diluted. The LOQ was determined at the signal-to-noise ratio (S/N) of about 10, and LOD was determined at the S/N ratio of 3. Intraday and interday experiments were carried out to test the repeatability and reproducibility of the method. The intraday variation was determined by six repetitive injections of the standard mixture on the same day, whereas the interday variation was determined by three repetitive injections of the standard mixture on three consecutive days. A recovery test was used to determine the accuracy of the method. An accurate quantity of standard solutions with three different concentration levels was added to 100 mg of sample and was further extracted and analyzed in triplicate. The average recoveries were determined by the formula: average recovery (%) = (detected amount – original amount)/spiked amount ×100.

### 2.9 *In vivo* studies

#### 2.9.1 Laboratory animals

Adult female Sprague Dawley (SD) rats (220 ± 20 g) were obtained from the National Laboratory Animal Centre, CSIR-CDRI. Animal care and experimental procedures were approved by the Institutional Animal Ethics Committee (Registration no.: 34/GO/ReBiBt-S/Re-L/99 CPCSEA) (IAEC/2020/60/renew/dated-03/01/2020). Animals were acclimatized for seven days before surgery, caged, and maintained at 22°C–25°C with 12-hour light/dark cycles. During the experimental period, the rats were maintained on a standard rodent chow diet and purified water ad libitum. Before surgery, animals were anesthetized using xylazine (10 mg/kg) and ketamine (40 mg/kg) injection (intramuscular).

#### 2.9.2 Osteotomy healing study

Forty-two female SD rats (220 ± 20 g, three months old, sexually mature) were taken, and drill holes of 0.8 mm were made at the femoral mid-diaphysis according to our previously published protocol (18, 19). Postsurgery, rats were divided randomly in the presence of two researchers into seven groups (*n* = 6 rat/group); vehicle (water), USKECSE (100 mg/kg, 150 mg/kg, 200 mg/kg), USCSE (100 mg/kg, 150 mg/kg, 200 mg/kg). All treatments were given orally daily for 12 days. All animals received a subcutaneous (s.c.) injection of calcein (20 mg/kg) 24 hours before sacrifice. For the calcein labeling studies, bones were embedded in acrylic material, and 60 μm sections through the osteotomy site were made using an Isomet-Slow Speed Bone Cutter (Buehler, Lake Bluff, IL) (19). Sections were photographed using a confocal microscope (Leica TCS SP-8, Wetzlar, Germany) and analyzed using LAS-X software.

#### 2.9.3 Studies on OVX rats

We next assessed the anti-osteoporosis effect of USKECSE in preventive mode using OVX rats. For this, 24 SD rats (220 ± 20 g, three months old) were either OVX bilaterally or sham-operated according to the previously published method (20). In brief, rats were anaesthetized with ketamine (40 mg/kg) and xylazine (10 mg/kg) and bilaterally OVX, whereas in the sham group ovaries were kept intact. Essential postoperative care was provided. After one week of surgery, the OVX rats were randomly divided in the presence of two researchers into two equal groups (*n* = 8 rat/group): OVX +Veh (water); OVX + USKECSE (100 mg/kg, oral) and the sham-operated animals received vehicle (water). All the treatments were given daily for 16 weeks. All animals received calcein (20 mg/kg) twice before sacrifice at 10-day intervals for dynamic histomorphometry (19).

Serum samples, bones (femur, tibia, and L5 vertebrae), and urine samples were collected after the treatment and stored at -80°C until further analysis.

#### 2.9.4 Body composition analysis

The body weight of all the groups in the study was taken once a week during the experimental period. The body composition of live rats was analyzed by the EchoMRI™-500 body composition analyzer (EchoMRI Corporation Pvt. Ltd. Singapore) 24 hours before the end of the experiment as per our previously described protocol (20). The machine was calibrated by scanning known samples of canola oil and distilled water before every scan. The total body weight and lean mass were plotted as obtained, whereas the fat mass was plotted by normalizing with the total body weight.

#### 2.9.5 Determination of biochemical measurements

Serum was also used to measure total bilirubin (DMSO, Dimethylsulfoxide method), alanine aminotransferase (ALT or SGPT) IFCC kinetic without P5P method, and aspartate aminotransferase (AST or SGOT) IFCC kinetic without P5P method. All of the parameters were measured using a fully automated clinical chemistry analyzer EM200/B160849 (TRANSASIA Bio-medical Ltd, India, Erba Manheim) by using supplier kits.

#### 2.9.6 μCT analysis

Scanning of bone samples was done by a SkyScan 1276 μ-computed tomography (μCT) scanner (SkyScan Ltd., Kartuizersweg, Kontich, Belgium) as described in our previously published protocol (20). Quantification of various bone parameters was performed by batman software (18). Reconstructed μCT images underwent a blind evaluation by a third person to determine the extent of bone loss.

#### 2.9.7 Serum PINP and urine CTXI level determination

A rat pro-collagen type I N-terminal propeptide (PINP) kit (Cat no: E-EL-R1414) and a cross-linked C-telopeptide of type I collagen (CTX-I) kit (Cat no: E-EL-R1456) were procured from Elabscience, USA, and measured by following the manufacturer’s protocol.

#### 2.9.8 L5 compression test

Bone mechanical strength was assessed by L5 compression test using a bone strength tester, TK 252C (Muromachi Kikai Co. Ltd. Tokyo, Japan) as described previously (21).

#### 2.9.9 *Ex vivo* mineralization assay

Bone marrow from rat femur was flushed by PBS, and cells were quantified by a hemocytometer. Bone marrow cells (2 × 10^6^) were seeded in a differentiation medium (α-MEM with 10 mM β-glycerophosphate, 50 μg/ml ascorbic acid, and 100 nM dexamethasone) in a six-well plate. The media was changed every 48 hours for 21 days. After 21 days, cultures were fixed with 4% formaldehyde, and mineralized nodules were visualized by staining with Alizarin red-S stain. The stain was extracted with 10% cetylpyridinium chloride (CPC), and the mineralization was quantified calorimetrically at OD 595 (22).

#### 2.9.10 Bone dynamic histomorphometry

Surface referent bone formation was analyzed by double calcein labeling as described in our previously published protocols (19, 23). Bioquant Osteo software (Bioquant Image Analysis, Nashville, TN) was used to measure mineralizing surface per bone surface (MS/BS), mineral apposition rate (MAR), and bone formation rate per bone surface (BFR/BS).

#### 2.9.11 Pharmacokinetic study

Female SD rats (*n*=6) were orally administered 100 mg/kg of USKECSE after overnight fasting. Subsequently, blood samples (approximately 0.2 mL) were collected at each time point, 0.25, 0.5, 1, 1.5, 2, 4, 6, 8, 12, 24, and 48 hours *via* minimal retro-orbital plexus in a 1.5-mL microcentrifuge tube containing sodium EDTA. Plasma was extracted from the blood samples by centrifugation at 8000 rpm for 10 minutes. The collected plasma samples were transferred into fresh tubes and then stored at -20°C until analysis.

The LC-MS/MS method was developed and samples were processed by using an API Q-TRAP 4000 mass spectrometer with an electrospray ionization (ESI) source. Analysis of the analyte (kaempferol) was performed by using multireaction monitoring (MRM) in the positive ion mode. Instrument parameters of both kaempferol (target analyte) and phenacetin (IS) were used, such as CUR: 30, CAD: high, ion source temperature (°C): 5500, vaporization temperature: 400°C, GS1 and GS2: 40 and 60. Whereas, compound parameters of kaempferol were precursor ion (m/z): 287.1, product ion (m/z): 153.2, DP (V): 106, CE:30, EP (V): 30, CXP (V): 14. For internal standard, phenacetin: precursor ion (m/z): 180.2, product ion (m/z): 110.2, DP (V): 71, CE:30, EP (V):10, CXP (V): 10. For chromatographic separation, X-bridge peptide column BEH C-18 (300A°, 250 mm x 4.6 mm, 5 μm) column with mobile phase of MeOH: 0.1% FA in TDW, 70:30 (v/v, %) was used. The compound was separated at a flow rate of 0.6 mL/min with a retention time of 6.2 minutes. Calibration standards (CS, 20-1000 ng/mL) were used for quantification of analyte and the liquid–liquid extraction (LLE) method was executed followed by protein precipitation (PPT).

Plasma samples (50 µl) were treated with 50 µl of 1 mg/mL of β-glucuronidase in 100 mM ammonium acetate buffer (pH-5) for two hours at 37°C. Subsequently, 200 µl ACN containing phenacetin (IS, 10 ng/mL) was added and gently vortexed for 20 seconds, and then, 2 mL ethyl acetate was added for separation of the compound from the plasma matrix and again vortexed for 10 minutes at 1000 rpm on a BenchMixer™. Samples were centrifuged at 10,000 rpm for five minutes, and supernatant (1.6 ml) was collected in a fresh RIA vial tube. After that, samples were completely dried on turbo Vap at 40°C temperature. The dried residue was reconstituted with 100 µl of methanol, and 10 µl was injected for LC-MS/MS analysis. Data were used for pharmacokinetic and statistical analysis, and the plasma concentration *vs.* time data was plotted and analyzed by noncompartmental analysis using Pheonix 6.3 WinNonlin (Pharsight Corporation, Mountain View, CA, USA). Different parameters, viz. C_max_ (ng/mL) - maximum plasma concentration; T_max_ (h) - time to reach C_max_; AUC0-∞ (ng h/mL) - area under concentration *vs.* time curve extrapolated to infinity; t½ (h) - elimination half-life; V_d_ (l) - volume of distribution; and Cl_r_ (L/h) - total clearance, were calculated (18, 24).

#### 2.9.12 Genotoxicity studies

A standard battery of tests was done to evaluate the genotoxicity of USKECSE according to the standard protocols as described in OECD (Organization for Economic Co-operation and Development) guidelines.

##### 2.9.12.1 *Ames assay*

USKECSE was tested for its mutagenic potential in the bacterial reverse mutation assay. The study was conducted using TA98, TA100, TA1535, and TA1537 tester strains of *Salmonella typhimurium* and WP2uvrA (pKM101) tester strains of *Escherichia coli* following our previously published protocol (25). The bacterial tester strains were exposed to USKECSE in triplicates at 50, 158, 500, 1581, and 5000 μg/plate. This study was conducted in accordance with the OECD Guideline No. 471 for testing of chemicals, “Bacterial Reverse Mutation Test” adopted July 21, 1997 (OECD, 1997) (26). The number of revertants was compared with controls and positive controls to draw the conclusion.

##### 2.9.12.2 *In vitro mammalian chromosomal aberration test*

This study was conducted in accordance with the OECD Guideline No. 473 for testing of chemicals, “*In vitro* Mammalian Chromosomal Aberration Test” adopted July 29, 2016 (OECD, 2016). Blood cells were exposed to USKECSE at 69, 208, and 625 µg/mL concentrations along with a DMSO control for three hours in the presence or absence of exogenous metabolic activation for 22 hours in the absence of metabolic activation. Following exposure, dividing lymphocytes were treated with colchicine to arrest the cells in a metaphase-like stage of mitosis (c-metaphase). Cells were then harvested, and metaphase preparations were made for chromosomal analysis. Preparations were stained with Giemsa, and metaphases were analyzed for chromosomal aberrations. One hundred fifty metaphases from each replicate culture were analyzed for chromosome aberrations.

##### 2.9.12.3 *In vivo mammalian bone marrow chromosomal aberration test*

This study was performed in accordance with the OECD Guidelines for the Testing of Chemicals, No. 475, “Mammalian Bone Marrow Chromosomal Aberration Test” adopted July 29, 2016. USKECSE was administered at three doses (500, 1000, and 2000 mg/kg/day) along with vehicle (5% ethanol + 10% PEG 400 + 0.5% w/v sodium carboxymethyl cellulose - medium viscosity containing 0.1% v/v Tween 80 in Milli Q water) and positive control (cyclophosphamide monohydrate, 15 mg/kg/day) twice at an interval of 24 hours by oral gavage to Swiss albino mice. The mice were sacrificed 24 hours after the test item administration. Before sacrifice, the mice were treated with a spindle inhibitor, 0.04% colchicine (10 ml/kg), to arrest the cells in metaphase. Chromosome preparations from the femur bone marrow cells were stained and scored for aberrations and the mitotic index (%) was determined. The frequency of mitotic divisions (mitotic index) was estimated by counting the number of metaphase plates per 1000 blast cells per animal. Slides were screened for 200 analyzable metaphases per animal and scored for aberrations.

#### 2.9.13 Acute toxicity study

The acute toxicity study in rats was carried out as per OECD Principles of Good Laboratory Practice [C(97)186/Final] and US FDA Good Laboratory Practice for Nonclinical Laboratory Studies (21 CFR Part 58). Healthy adult SD rats (*n* = 25 males and 25 females) were recruited. Rats were divided into five groups in each sex and given oral gavage of vehicle (1% gum acacia in water) and USKECSE (500 mg/kg, 1000 mg/kg, 1500 mg/kg, and 2000 mg/kg). The behavior of animals was observed for 14 days after which all groups were terminated to observe the gross pathology.

#### 2.9.14 *In vitro* studies

##### 2.9.14.1 *Rat calvarial osteoblast culture and ALP assay*

Rat calvarial osteoblasts (RCO) were cultured from one- to two-day-old rat pups as described before in our previously described protocol (19). At 90% confluence, cells were trypsinized and seeded in 96-well plates to obtain adherent cells for ALP assay. These cells were treated with either vehicle or kaempferol (2 µM) for 48 hours in a differentiation medium (α-MEM supplemented with 10 mM β-glycerophosphate and 50 μg/mL ascorbic acid). After 48 hours, diethanol amine buffer (DAE) with 2 mg/ml para-nitrophenyl phosphate (pNPP) was added to measure ALP activity colorimetrically at OD 405 nm.

#### 2.9.15 Quality control for all studies

All the studies conducted and data collected were checked by minimum of two observers. Critical phases of the study plans as well as any amendments were applied to the test site in accordance with GLP compliance management guidelines, and the audited raw data were deposited with the senior author.

#### 2.9.16 Statistical analyses

Data are expressed as the mean ± standard error of the mean (SEM). Statistical differences among the different treatment groups were analyzed by one-way ANOVA followed by a *post hoc* Tukey test using GraphPad Prism 5 with a significance level of 0.05% (95% significance).

## 3 Results

### 3.1 Optimization of extraction and hydrolysis conditions

To achieve the optimal extraction of the target compound, different extraction solvents [*n*-hexane, EtOAc methanol, ethanol, methanol-water (4:1, 1:1) and ethanol-water (4:1, 1:1), ethanol-EtOAc (4:1, 1:1)], and extraction techniques (percolation, sonication, and reflux) were optimized. The developed UPLC method was applied for the quantification of kaempferol in different samples. Extraction by repeated percolation technique [*n*-hexane→ EtOAc→ ethanol-water (4:1)] was found to be the best method for the highest extraction of the target compounds.

To achieve the optimal hydrolysis of kaempferol glycosides present in the extract, solvents such as methanol-water (1:1) and ethanol-water (1:1), the quantity of solvent (60 ml and 100 ml), hydrolysis conditions such as concentration of acid (2 M, 4 M, and 6 M), and quantity of acid (15 ml and 25 ml) were optimized under reflux conditions and were analyzed by UPLC. One gram of extract refluxed with 2 M HCl (2 mL) in methanol-water (1:1, 10 ml) for three hours provided the best condition for the enrichment of kaempferol. These optimized conditions were further applied to enrich kaempferol in *C. sinensis* hydroethanolic extract at a 220 g scale. *C. sinensis* hydrolyzed extract (acidified) thus obtained from the hydrolysis experiment was treated with 220 g of Amberlyst A-21 free base resin in MeOH: H_2_O (1:1, 1000 ml) at 50°C for three hours under constant stirring (**Figure 1**).

### 3.2 Optimization of chromatographic conditions

**Figure 2:**
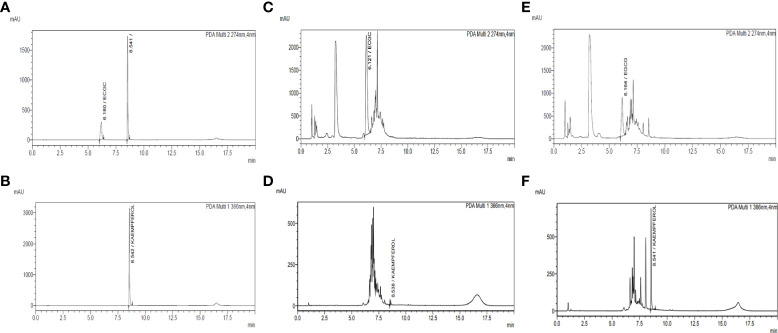
Quantification of EGCG and kaempferol by UPLC-PDA in *C. sinensis* hydroethanolic extract and kaempferol-enriched *C. sinensis* extract. **(A)** Chromatogram of the standard mixture of kaempferol and EGCG at 274 nm. **(B)** Chromatogram of the standard mixture of kaempferol and EGCG at 366 nm. **(C)** Chromatogram of *C. sinensis* hydroethanolic extract (USCSE) at 274 nm. **(D)** Chromatogram of *C. sinensis* hydroethanolic extract (USCSE) at 366 nm. **(E)** Chromatogram of kaempferol enriched *C. sinensis* extract (USKECSE) at 274 nm. **(F)** Chromatogram of kaempferol enriched *C. sinensis* extract (USKECSE) at 366 nm.

The UPLC conditions were optimized by using a standard mixture of the compounds and the *C. sinensis* hydroethanolic extract. Different mobile phases, such as acetonitrile-water (0.1% and 0.05% formic acid), methanol-water, acetonitrile-water, and column temperatures (25°C, 27°C, and 30°C), were optimized for the accurate detection of standard compounds in the UPLC analysis. The best separation was obtained under a low-pressure gradient with a flow rate of 0.240 mL/min from acetonitrile-water (0.1% formic acid) at a 27°C column temperature. The optimized conditions yielded reproducible retention time and symmetric peak shape (**Figure 2**).

### 3.3 UPLC method validation for quantitative analysis

The compounds showed a coefficient of determination (*r* ^2^) in the range of 0.99, which indicates good linearity of the developed method. The LOD for the two analytes was observed in the range of 0.03–0.83 μg/mL, whereas the LOQ was observed in the range of 0.09–2.5 μg/mL. The RSD value of intraday (*n* = 6) and interday (*n* = 3) precisions were found to be in the range of 0.4% and 4.4%, respectively. The recoveries of both analytes were found to be in the range of 89.0%–97.9% with RSD ranging from 0.07% to 1.08% (**Table 1**). The results demonstrate that the developed method is sensitive, precise, and accurate for the quantitative analysis of the intended compounds in *C. sinensis* extract.

**Table 1:** Regression equation, linear range, LOD, LOQ, intra-day, inter-day and recovery studies for the target analytes.

Analyte	Regression equation^a^	R^2^	Linear range (µg/mL)	LOD^b^	LOQ^c^	Intra- day^d^ RSD (%)n = 6	Inter-day RSD (%)n = 3	Recovery^e^				
								Original(µg)	Spiked(µg)	Detected(µg)	Average recovery (%)	RSD (%)
EGCG	Y= 4847.90X + 7970.49	0.9945	2.5 - 320	0.83	2.5	4.46	0.40	5567.4	96	5654.6	90.7	0.10
									120	5674.3	89.0	0.27
									144	5708.5	97.9	0.30
Kaempferol	Y= 16892.4X -2790.77	0.9998	0.09 -31.25	0.03	0.09	3.16	1.20	7.7	48	54.6	97.6	1.08
									60	66.01	97.0	0.07
									72	77.36	96.6	0.90

a The regression equations were presented as Y = mX + c. Y and X were defined as peak area and concentration of compound, respectively.

b LOD, limit of detection, S/N = 3.

c LOQ, limit of quantification, S/N = 10.

d Intra and inter-day precision was determined on the basis of peak area. RSD (%) = (SD/mean) × 100.

e Average recovery (%) = (detected amount − original amount)/spiked amount × 100.

### 3.4 Qualitative and quantitative analysis of samples

The developed UPLC-PDA method was applied for simultaneous quantification of two target analytes in *C. sinensis* hydroethanolic extract (USCSE) and kaempferol-enriched *C. sinensis* extract (USKECSE). The chromatograms for the standard mixture and samples are presented in **Figure 2**. Different samples were analyzed for the quantification of standard compounds in the optimization experiments. As shown in **Table 2**, kaempferol content in two different samples ranged from 0.2 to 7.8 mg/g. With 7.8 mg/g, USKECSE has higher kaempferol content than USCSE (0.2 mg/g). Of the two analyzed compounds, kaempferol was enriched in the samples after the acid hydrolysis procedure, whereas the EGCG amount decreased from 232.2 mg/g to 93.4 mg/g. These data demonstrate enrichment of kaempferol in the *C. sinensis* extract, i.e., USKECSE was achieved by developing a precise and accurate analytical method for the simultaneous quantification of kaempferol and EGCG *via* UPLC-PDA.

**Table 2:** Amount (mg/g) of target analytes in *C. sinensis* hydroethanolic extract (USCSE) and kaempferol enriched *C. sinensis* extract (USKECSE).

Sample code	EGCG	Kaempferol
Hydroethanolic extract (USCSE)	232.2 ± 6.6	0.2 ± 0.2
Kaempferol enriched extract (USKECSE)	93.4 ± 3.9	7.8 ± 0.8

### 3.5 Bone regenerative effect of kaempferol-enriched USKECSE in osteotomy model

**Figure 3:**
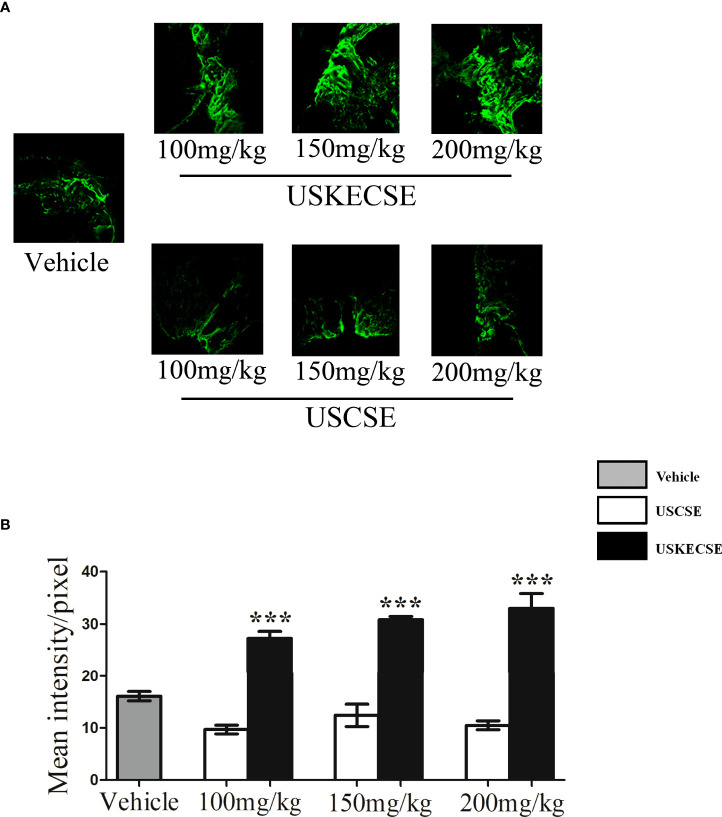
USKECSE promoted bone regeneration at the femur osteotomy site. Adult female rats were treated with vehicle, USCSE, or USKECSE at indicated doses 24 hours after femur osteotomy and continued for 12 days. **(A)** Representative images of calcein deposition at the osteotomy site and **(B)** quantification of calcein by confocal microscopy. Values are expressed as mean ± SEM (*n* = 6 bones/group); ***p<.001 vs. vehicle.

We first tested the osteogenic effect measured by bone regeneration at the osteotomy site of rats treated with USCSE and USKECSE at 100-, 150-, and 200-mg/kg doses. Compared with vehicle-treated rats, USKECSE at all doses significantly increased calcein intensity (nascent bone formation). USCSE, however, had no effect at any of the doses tested (**Figures 3A, B**). Because the 100-mg/kg dose of USKECSE showed a significant bone regenerative effect, we selected this dose as the minimum effective dose in our further studies.

### 3.6 Effect of USKECSE on body composition and liver function in OVX rats

**Figure 4:**
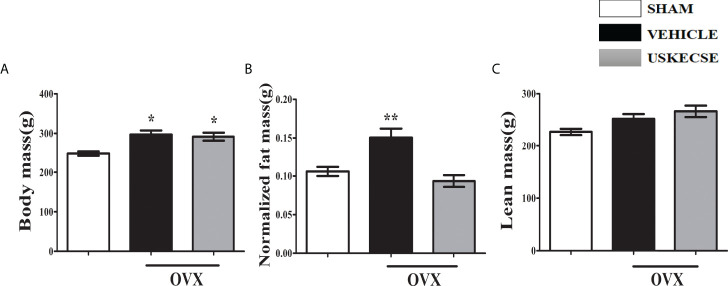
USKECSE decreased fat mass in osteopenic rats. **(A)** Body mass, **(B)** normalized fat mass, and **(C)** lean mass were measured. For parameters shown in **(B)** and **(C)**, Echo-MRI was used. Values are expressed as mean ± SEM (*n* = 6 rats per group); *p <.05, **p <.01, vs. sham.

OVX increased body weight compared with control, and USKECSE had no effect on the OVX-induced body weight in rats (**Figure 4A**). Fat mass was significantly increased in OVX rats compared with control, but it was comparable between the USKECSE and control groups (**Figure 4B**). Lean mass was, however, comparable across the groups (**Figure 4C**).

**Figure 5:**
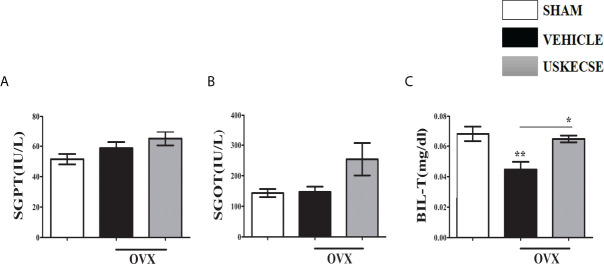
USKECSE has no hepatotoxicity. **(A)** Serum glutamic pyruvic transaminase (SGPT), **(B)** serum glutamic oxaloacetic transaminase (SGOT), and **(C)** serum total bilirubin (T-Bil) data in the indicated groups are shown. Values are expressed as mean ± SEM (*n* = 6 serum samples per group); *p <.05, **p <.01, vs. sham.

Given the reported hepatotoxicity in *C. sinensis* extract (11, 27), we assessed the liver function of OVX rats after 16 weeks of USKECSE treatment. SGPT (ALT) and SGOT (AST) were comparable across the groups (**Figures 5A, B**). Total bilirubin (Bil-T) was significantly decreased in OVX compared with sham, whereas USKECSE treatment brought the Bil-T levels to the sham levels (**Figure 5C**). The results suggest that USKECSE has no hepatotoxicity.

### 3.7 USKECSE maintained bone volume and microarchitecture of trabecular bones in OVX rats

**Figure 6:**
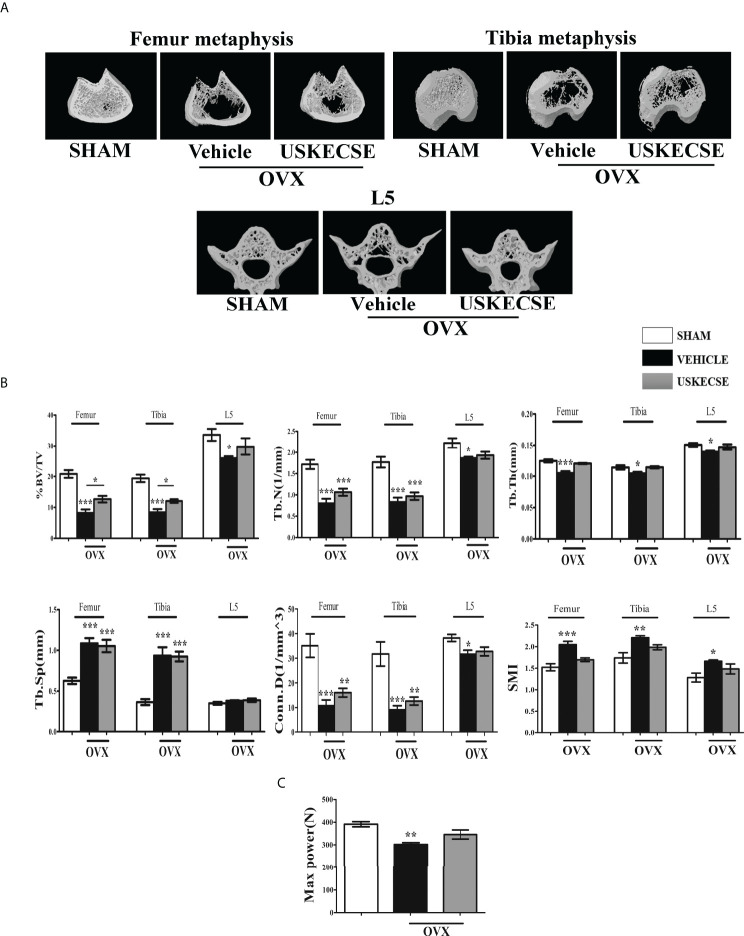
USKECSE prevented bone loss in osteopenic rats. **(A)** Representative images of femur metaphysis, tibia metaphysis, and L5 vertebrae are shown. **(B)** Shown are the quantitative µCT parameters of the femur, tibia metaphysis, and L5. %BV/TV, percent bone volume per tissue volume; Tb.N, trabecular number; Tb.Th, trabecular thickness; Tb.Sp, trabecular spacing; Conn.D., connectivity density; and SMI, structure model index. **(C)** The L5 compression strength was determined by a bone strength tester. Values are expressed as mean ± SEM (*n* = 6 bones/group); *p <.05, **p <.01, and ***p<.001 vs. sham.

Trabecular bones at femur metaphysis, tibia metaphysis, and L5 were studied using μCT (**Figure 6A** for representative images). Bone volume (BV/TV%) was significantly reduced in the OVX compared with control at all three sites and USKECSE treatment to OVX rats increased it at all sites compared with OVX rats given vehicle. Tb.N and Tb.Th were significantly decreased at all three sites studied in OVX rats compared with control, and USKECSE maintained these parameters to the sham level only at L5. Tb.sp was comparable across all groups in L5, and the OVX-induced increase in Tb.sp at femur and tibia remained unchanged by USKECSE treatment (**Figure 6B**).

The topological parameters, including connectivity density (Conn.D.) and structural model index (SMI), were significantly altered in OVX rats compared with control. Conn.D was reduced in OVX, and USKECSE-treated rats showed a significantly higher value than OVX rats. OVX rats displayed significantly higher SMI at the femur and L5, and USKECSE treatment maintained the values comparable to control (**Figure 6B**).

Furthermore, we studied biomechanical strength at L5 by applying compression force, which showed a significant decrease in maximum power in OVX rats, but USKECSE maintained it to the sham level (**Figure 6C**). Our study in OVX rats suggests that the osteoprotective property of USKECSE was more effective at L5 than femur and tibia. We next assessed the osteoanabolic effect of USKECSE.

### 3.8 USKECSE stimulates bone formation in OVX rats

**Figure 7:**
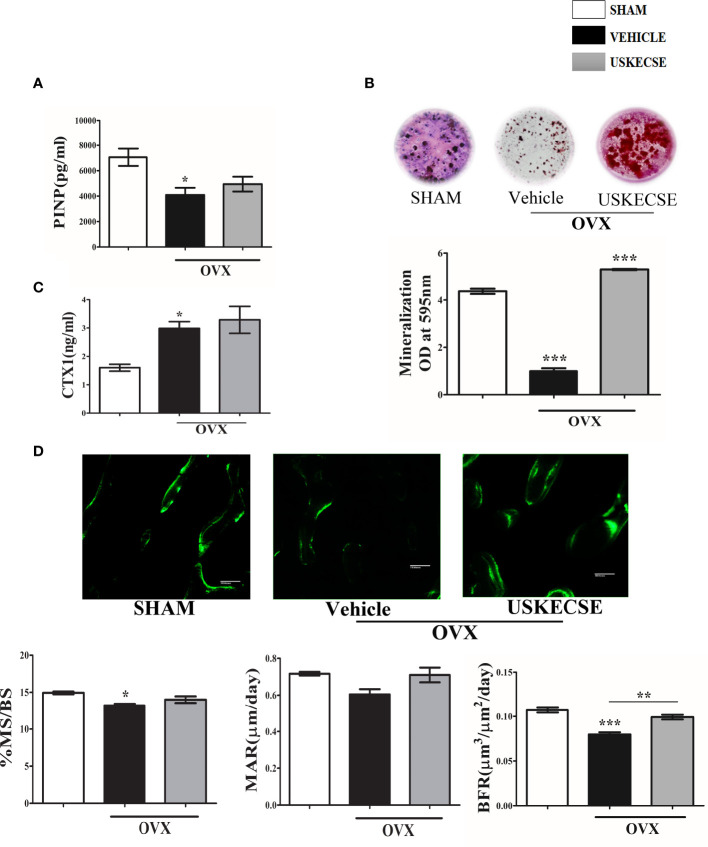
USKECSE has an osteoanabolic effect in osteopenic rats. **(A)** Serum procollagen type I N-propeptide (PINP). **(B)** *Ex vivo* mineralization assay was performed in samples obtained from the indicated groups. **(C)** Cross-linked C-telopeptide of type I collagen (CTX1) levels were determined by ELISA from the urine of rats with indicated treatments. **(D)** Upper panel showing representative images (scale bar, 100 μm) of double calcein labeled surface at femur metaphysis and the lower panel showing dynamic histomorphometry parameters in the indicated groups. For ELISA, serum samples of *n* = 6 rats from each group were taken, and for *ex vivo* mineralization and histomorphometry femurs, sections of *n* = 3 rats from each group were used. Values are expressed as mean ± SEM; *p <.05, **p <.01, and ***p<.001 vs. sham.

Serum PINP (an osteogenic marker) was reduced in OVX rats compared with sham, and USKECSE treatment maintained it to the sham level (**Figure 7A**). In the *ex vivo* nodule formation (mineralization) assay, we observed a sharp decrease in mineralization in the OVX group compared with the sham group. USKECSE-treated OVX rats displayed mineralization significantly higher than the sham (**Figure 7B**). Urine CTX-1 (a resorption marker) was significantly elevated in OVX rats compared with control, and USKECSE treatment failed to reduce the OVX-induced increase (**Figure 7C**).

The surface-referent bone formation measurement was studied, and we observed a decrease in MS/BS and BFR in OVX rats, and USKECSE treatment significantly increased both over the OVX group (**Figure 7D**).

### 3.9 Pharmacokinetic study

**Figure 8:**
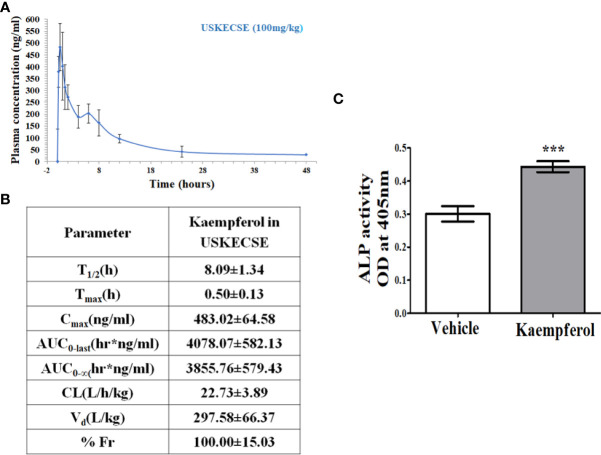
Kaempferol has oral bioavailability and an osteogenic effect upon USKECSE treatment. **(A)** Time-concentration profile of kaempferol in plasma after a single oral dose (100 mg/kg) of USKECSE in adult female rats. **(B)** Table showing calculated pharmacokinetic parameters. **(C)** Rat calvarial osteoblasts (RCO) were treated with 2 µM kaempferol, and differentiation was assessed by alkaline phosphatase (ALP) assay. Values are expressed as mean ± SEM; ***p <.001 vs. vehicle.

After a single oral dose (100 mg/kg) of USKECSE, kaempferol was detectable for 48 hours in the plasma as shown in the time-concentration plot (**Figure 8A**), suggesting its significant absorption and distribution. From the time-concentration plot, various PK parameters were determined as shown in **Figure 8B**.

Based on the C_max_ of kaempferol, the molar concentration was calculated to be ~2 μM. Cultured osteoblasts treated with 2 μM kaempferol promoted osteogenic differentiation (**Figure 8C**).

### 3.10 Genotoxicity of USKECSE

In mammalian cells, kaempferol is reported to have a genotoxic effect likely mediated by its P450-dependent biotransformation to quercetin (28, 29). In prokaryotic cells, through the Ames assay, kaempferol has been reported to have a direct genotoxic effect (30). In view of these reports, we assessed the genotoxicity of USKECSE in prokaryotic and eukaryotic cell systems in a GLP-accredited laboratory.

USKECSE ranging from 50 to 5000 µg/plate showed no bacterial mutagenicity assessed by the Ames test. The tested doses showed no positive mutagenic increase in the mean number of revertant colonies for all tester strains when compared with the respective vehicle control plates either in the presence or absence of the metabolic activation. The results indicate that USKECSE is not mutagenic (**Supplementary Tables 1, 2**, and **3** and its corresponding raw data in **Appendices 1, 2**, and **3**, respectively).

Next, we tested USKECSE in the clastogenic assay *in vitro* using human peripheral blood lymphocytes, and chromosomal aberrations were analyzed. Briefly, the human lymphocytes in the whole blood culture were first stimulated to divide by the addition of phytohemagglutinin (PHA) 48 hours before treatment and then were exposed to USKECSE in the presence and absence of an exogenous metabolic activation system (S9 fraction prepared from Aroclor 1254 induced rat liver). We first tested the lymphocyte cytotoxicity in response to USKECSE at 69, 208, and 625 μg/ml concentrations, and at the highest concentration, it caused cytotoxicity both in the presence and absence of metabolic activation (**Supplementary Tables 4** and **5** and its corresponding raw data in **Appendices 4** and **5**, respectively), and hence, 625 μg/ml was considered to be the maximum concentration for the chromosomal aberration assay. Subsequently, blood cultures were treated with USKECSE in duplicate at the aforementioned concentrations in the presence and absence of metabolic activation for three hours and also in the absence of metabolic activation for 22 hours (**Supplementary Table 6** and its corresponding raw data in **Appendix 6**). We used similar experimental conditions for vehicle control (DMSO) and the positive controls (cyclophosphamide monohydrate in the presence of metabolic activation and ethyl methanesulfonate in the absence of metabolic activation). Cells in all experimental groups were harvested at the C-metaphase stage at the end of the indicated treatments. A total of 300 metaphases from duplicate cultures from each treatment group were evaluated. The data from the treatment groups and the positive controls were statistically compared with the vehicle control. The mitotic index was reduced by ~45%. There was no statistically significant increase in the incidence of structurally aberrant chromosomes in the metaphases either in the presence or absence of metabolic activation in any of the tested concentrations of USKECSE. The respective positive controls showed statistically significant (*p* <.05) increases in aberrant metaphase chromosomes, confirming the sensitivity of the test system and the activity of the S9 mix (**Supplementary Tables 4–6**). The results suggest that USKECSE was not clastogenic on peripheral human blood lymphocytes up to the highest concentration (625 μg/ml) tested.

We next tested the clastogenicity of USKECSE in Swiss albino mice as it provides a rational basis for mutagenic risk assessment in humans. USKECSE was given oral administration (500, 1000, and 2000 mg/kg) for two consecutive days. An additional group of mice were given the positive control cyclophosphamide monohydrate (15 mg/kg) (**Supplementary Table 7**). The incidences of individual aberrations and percentage of mitotic index in bone marrow smears of males and females were comparable to the corresponding vehicle control group at all the doses of USKECSE (**Supplementary Tables 8** and **9** and the corresponding raw data in **Appendices 7 and 8**, respectively). By contrast, cyclophosphamide monohydrate treatment resulted in a significant increase in the percentage of cells with structural chromosomal aberrations (including and excluding gaps) and a decrease in mitotic index compared with vehicle controls. The results indicate that USKECSE was nonmutagenic in male and female mice at a >10X osteogenic dose.

### 3.11 Acute toxicity study

USKECSE was orally administered to adult rats at 500-, 1000-, 1500-, and 2000-mg/kg doses. None of the rats showed any adverse clinical signs at the time of sacrifice on the 15th day. This study suggests that the maximum tolerated dose of USKECSE is >2 g/kg.

## 4 Discussion

Green Tea Polyphenol (GTP) protected OVX and orchidectomized rats from trabecular bone loss (31, 32), where EGCG was the major polyphenol. However, in these studies, GTP was given in drinking water, thus precluding accurate estimation of the dose. Besides this, systemic administration of EGCG (3.4 mg/kg) has a bone-conserving effect in OVX rats (33). At the molecular level, EGCG activates the cellular nutrient sensor AMP-activated protein kinase (AMPK) in osteoblasts resulting in the downstream activation of mitochondrial biogenesis that, in turn, stimulates osteoblast differentiation (34). However, green tea–associated liver disease, including acute and fulminant liver damage, has been attributed to EGCG (35–38). By contrast, kaempferol showed a hepatoprotective effect in various preclinical models of liver damage (39–42) and, at the same time, has beneficial effects in bone (13, 43, 44). The anti-osteoporosis effect of kaempferol is mediated by various mechanisms in bone cells, including modulation of estrogen receptors, activation of BMP2 signaling, increasing the molecular interaction of TAZ (transcriptional coactivator with PDZ-binding motif) and Runx2 (osteoblast transcription factor) to augment the transcriptional activity of Runx2, inhibition of NF-κB and mTOR pathways, and inhibition of autophagy through downregulation of p62/SQSTM1 (45–47). Therefore, we developed USKECSE from *C. sinensis* extract by using a novel acid hydrolysis procedure to harness the salutary effect of green tea extract in bones by enhancing kaempferol and concomitant depletion of EGCG for liver safety. We observed that the kaempferol-enriched USKECSE promoted bone formation at the osteotomy site and, in OVX rats, maintained bone mass, microarchitecture, and bone strength solely by an osteoanabolic mechanism. Finally, USKECSE displayed no hepatotoxicity, genotoxicity, and systemic toxicity.

We compared the bone regeneration efficacy of USKECSE and its unformulated version, USCSE, in a rat femur osteotomy model to first determine the osteogenic dose. The osteogenic property of the extracts was determined by the amount of callus formation at the osteotomy site by calcein labeling (18, 48). We found that USKECSE (100 mg/kg) but not USCSE promoted bone regeneration. We next used 100 mg/kg USKECSE to study its effect on the development of osteopenia in OVX rats. Previous studies show that OVX induces gain in fat mass and body weight (20). Our data show that USKECSE treatment prevented fat mass gain to maintain it to the level of the control group. These results are consistent with a previous report that observed a significant decrease in body fat mass in visceral fat-type obesity in Japanese men and women by green tea extract (49). Increased kaempferol in USKECSE may also have contributed to reduction in fat mass as a kaempferol-rich extract of *Rhizoma Polygonati falcatum* (50) and pure kaempferol inhibited adipogenesis, and the latter effect was likely mediated by peroxisome-proliferated activator receptor-α (PPARα) (51). In our study, the liver function of OVX rats treated with USKECSE was comparable to control, which suggests that the formulation was devoid of hepatotoxicity.

μCT analysis of trabecular bones showed that USKECSE maintained bone mass, microarchitecture, and bone strength of the trabecular bones in OVX rats solely by an osteoanabolic mechanism. μCT data revealed that the preservation of trabecular bone volume and the integrity of the trabecular network by USKECSE was attributable more to the preservation of Tb.Th than Tb.N at the femur and tibia, whereas at L5, both Tb.Th and Tb.N were maintained to the sham levels. Conn.D represents the integrity of the trabecular network and is a measure of intact branches, whereas SMI represents rod and plate structures in trabecular bone determined by surface structures such that, for spherical, cylindrical rods and planar surfaces for which the values are 4, 3, and 0, respectively (52). Hence, higher SMI values indicate more spherical or cylindrical structures that are less stable, and lower values indicate more stable planar structures (53). In OVX rats, Conn.D. was reduced, which indicates broken trabecular branches, and USKECSE-treated rats showed a significantly higher value than OVX rats, indicating maintenance of intertrabecular connectivity. SMI at femur and L5 was significantly higher, whereas USKECSE treatment maintained values comparable to control. Taken together, from these 3-D topological parameters, it appears that the trabecular bone of OVX rats would have less buckling strength than the control, and USKECSE significantly maintained these, particularly at L5. Indeed, measurement of compression strength at L5 showed that USKECSE maintained the strength in OVX rats comparable to the control. Osteoporotic vertebral compression fractures affect ~25% of all postmenopausal women (54), and our study demonstrates that the bone-conserving and strength-maintenance effects of USKECSE were most effective at L5 of OVX rats. The first line of osteoanabolic therapy, teriparatide acting *via* the type 1 parathyroid hormone receptor, was found to be more efficient in inhibiting vertebral fracture than hip fracture (69% vertebral fracture vs. 54% hip fracture reduction) (55). The action of USKECSE was reminiscent of teriparatide’s action as it was more effective in improving L5 parameters than femur.

The serum PINP was maintained in the OVX +USKECSE group comparable to the sham level, whereas USKECSE treatment had no effect on urine CTX-1. *Ex vivo* nodule formation data clearly displays that USKECSE treatment promoted mineralization higher than the sham. The surface-referent bone formation measurement, which is considered the most reliable determinant of the osteoanabolic effect in response to any treatment, showed that both MS/BS and BFR in the OVX + USKECSE group were significantly increased compared with OVX rats. These data suggest that USKECSE is a pure osteoanabolic agent. In the future, the effects of USKECSE in a therapeutic regimen (OVX rats with established osteopenia) are required to be investigated to firmly establish it as an osteogenic agent before evaluating it in postmenopausal osteoporosis patients. In this regard, it would be interesting to combine USKECSE with alendronate, a widely used oral bisphosphonate having a strong antiresorptive effect to assess whether the combination therapy is better than the monotherapy. Besides EGCG, green tea contains other catechins, including epigallocatechin, gallocatechin, and gallocatechin gallate, having beneficial effects on bone (56). Our process of enriching kamepferol in USKECSE, although reduced in EGCG (an osteogenic compound), however, likely retained other catechins with salutary effects on bone. Thus, a combination of a high amount of kaempferol and tea catechins afforded a dual advantage of inducing osteogenic response and eliminating the hepatotoxic effect of EGCG.

A PK study of a single oral dose (100 mg/kg) of USKECSE suggests significant absorption and distribution of kaempferol, and the profile was similar to that observed with pure kaempferol in adult rats (13). Based on the C_max_ of kaempferol, the molar concentration was calculated to be ~2 μM. At this concentration, kaempferol significantly increased osteogenic differentiation of cultured osteoblasts, which suggests that the serum level of the compound achieved by 100 mg/kg USKECSE was functional as it was sufficient to induce osteogenic response.

Genotoxicity of USKECSE was assessed in prokaryotic, eukaryotic cells, and in Swiss albino mice. In the Ames test, USKECSE showed no mutagenic effect, and in human peripheral blood lymphocytes, USKECSE was not clastogenic up to the highest concentration tested. Similarly, in male and female mice, USKECSE showed no mutagenic effects. In an acute toxicity study, the therapeutic index of USKECSE was derived >20-fold, which suggests the safety of the extract.

## Conclusions

A formulation, USKECSE, developed from tea extract by a novel extraction procedure enhanced kaempferol content and concomitantly depleted the hepatotoxic compound, EGCG. USKECSE is more potent than the hydroethanolic extract in terms of the beneficial skeletal effects and is devoid of hepatotoxicity. Kaempferol in USKECSE has significant oral bioavailability that translates to an osteogenic effect. Safety studies reveal that USKECSE has a 20X therapeutic index and has no genotoxicity. Based on these findings, we propose a pilot clinical trial of USKECSE in postmenopausal osteoporosis for enhancing the evidence base toward positioning this novel extract as a nutraceutical supplement for bone health.

## Data availability statement

The raw data supporting the conclusions of this article will be made available by the authors, without undue reservation.

## Ethics Statement

The animal study was reviewed and approved by Institutional Animal Ethics Committee, National Laboratory Animal Centre, CSIR-CDRI (Registration no.: 34/GO/ReBiBt-S/Re-L/99 CPCSEA) (IAEC/2020/60/renew/dated-03/01/2020).

## Author contributions

NC, CK, and SS conceptualized the idea of manuscript. CK and SS performed major experiments. SR and KP helped in the osteotomy and OVX surgery. NC and CK wrote and evaluated the manuscript. μCT of bones and calcein labeling assessment in osteotomy study was done by CK and SS. μCT of bones in the OVX study and its analysis related to trabecular bone parameters, bone strength test experiment, body composition analysis, liver function test were conducted by CK. Bone dynamic histomorphometry, *ex-vivo* mineralization and ELISA were performed and analyzed by SS. Pharmacokinetic study was done by JRG and SV. US and PSB done the extract preparation and UPLC. SKR carried out toxicity experiments. All authors concur to the final version of the manuscript.

## Funding

NC and US acknowledge grant-in-aid support from CSIR, CSIR-Nutraceutical Mission Project (HCP0019-2.1).

## Acknowledgments

The assistance provided by Dr. Shail Singh, Division of Toxicology & Experimental Medicine in conducting Liver Function Test is greatly appreciable. Authors acknowledge Rima Ray Sarkar, confocal facility, Division of Molecular and Structural Biology, CSIR-CDRI, for her assistance with confocal imaging. Upendra Sharma thanks CSIR for financial support under CSIR-Nutraceutical Mission. The CDRI communication number for this paper is 10429.

## Conflict of interest

The authors declare that the research was conducted in the absence of any commercial or financial relationships that could be construed as a potential conflict of interest.

## Publisher’s note

All claims expressed in this article are solely those of the authors and do not necessarily represent those of their affiliated organizations, or those of the publisher, the editors and the reviewers. Any product that may be evaluated in this article, or claim that may be made by its manufacturer, is not guaranteed or endorsed by the publisher.
