# Supplementary material for: A novel extraction method enhanced the osteogenic and anti-osteoporosis effect of tea extract without any hepatotoxicity in ovariectomized rats
Source: Front Endocrinol (Lausanne). 2022 Aug 8;13:951800. doi: 10.3389/fendo.2022.951800 (PMC9434695; doi:10.3389/fendo.2022.951800)
Supplement: Supplementary file 1 [file DataSheet_1.docx]

**Supplementary Materials**

**A novel extraction method enhanced the osteogenic and anti-osteoporosis effect of tea extract without any hepatotoxicity in ovariectomized rats**

Chirag Kulkarni^1,2†^, Shivani Sharma^1,2†^, Prateek Bora^3^, Saurabh Verma^2,4^, Swati Rajput^1,2^, Konica Porwal^1^, Srikanta Kumar Rath^2,5^, Jiaur Rahaman Gayen^2,4^, Upendra Sharma^3^, Naibedya Chattopadhyay^1,2*^

^1^Division of Endocrinology and Centre for Research in Anabolic Skeletal Targets in Health and Illness (ASTHI), CSIR-Central Drug Research Institute, Lucknow, India

^2^Academy of Scientific and Innovative Research (AcSIR), Ghaziabad- 201002, India

^3^Division of Chemical Technology, CSIR-Institute of Himalayan Bioresource Technology, Palampur, India

^4^Division of Pharmaceutics & Pharmacokinetics, CSIR-Central Drug Research Institute, Lucknow, India

^5^Division of Toxicology and Experimental Medicine, CSIR-Central Drug Research Institute, Lucknow, India

^†^These authors have contributed equally to this work

*, To whom correspondence should be addressed: Naibedya Chattopadhyay Endocrinology Division, CSIR-Central Drug Research Institute, Sector 10, Jankipuram Extension, Sitapur Road, Lucknow–226 031, India, Tel: 091-522-2771940 (Ext.-4967); Fax: 091-522-2771941, E-mail: [n_chattopadhyay@cdri.res.in](mailto:n_chattopadhyay@cdri.res.in).

**Material and methods**

**1 Genotoxicity**

**1.1 Ames assay**

The study was done as per OECD Principles of Good Laboratory Practice according to OECD Guideline No. 471 for testing of chemicals, “Bacterial Reverse Mutation Test”, adopted: July 21, 1997 (OECD, 1997).

**Controls**

**Vehicle Control**

One hundred microliters of DMSO (100 μL) was used as the vehicle control.

**Positive Controls**

DMSO was used to prepare the stock and dilutions of the following positive controls

| **Strain** | **Activation** | **Positive controls (CAS No.)** | **Dose (µg/plate)** |
| --- | --- | --- | --- |
| TA98 | + | 2-Aminoanthracene (613-13-8) | 4 |
|  | - | 2-Nitrofluorene (607-57-8) | 2 |
| TA100 | + | 2-Aminoanthracene (613-13-8) | 4 |
|  | - | Sodium azide (26628-22-8) | 1 |
| TA1535 | + | 2-Aminoanthracene (613-13-8) | 4 |
|  | - | Sodium azide (26628-22-8) | 1 |
| TA1537 | + | 2-Aminoanthracene (613-13-8) | 4 |
|  | - | 9-Aminoacridine (90-45-9) | 50 |
| WP2*uvr*A  (pKM101) | +  - | 2-Aminoanthracene (613-13-8)  4-Nitroquinoline-1-oxide (56-57-5) | 30  4 |

| **Positive Control** | **Batch/Lot No.** | **Manufactured by** |
| --- | --- | --- |
| 2-Nitrofluorene | S43858V | Sigma Aldrich St. Louis USA |
| 2-Aminoanthracene | STBG0630V |  |
| 9-Aminoacridine | BCCB4167 |  |
| 4-Nitroquinoline-1-Oxide | WXBC1554V |  |
| Sodium azide | BCBJ9887V |  |

**Test System**

Following strains of bacteria approved under the 1997 OECD guideline for the assessment of point gene mutation were used:

Histidine auxotrophic strains of *Salmonella typhimurium*: TA98, TA100, TA1535 and TA1537.

Tryptophan auxotrophic strain of *Escherichia coli*: WP2*uvr*A (pKM101).

**Source of the Test System**

| ***Salmonella typhimurium*** | ***Escherichia coli*** |
| --- | --- |
| Health Protection Agency National Collection of Type Cultures (NCTC)  61, Colindale Avenue London NW9 5EQ Great Britain | The National Collection of Industrial and Marine Bacteria Ltd. (NCIMB) Ferguson Building  Craibstone Estate, Bucksburn Aberdeen, AB21 9YA Scotland, U.K. |

**Genotypic Characterization of Test System**

| **Genotype** | **TA 98** | **TA 100** | **TA 1535** | **TA 1537** | **WP2*uvr*A (pKM101)** |
| --- | --- | --- | --- | --- | --- |
| Tryptophan/ Histidine  Requirement | His + | His + | His + | His + | Trp + |
| *rfa* mutation | + | + | + | + | Not applicable |
| *uvr*B/*uvr*A  mutation | *uvr*B | *uvr*B | *uvr*B | *uvr*B | *uvr*A |
| R-factor | + | + | Not applicable | Not applicable | + |

**Test Medium and Solutions**

| 1 | VB agar |
| --- | --- |
| 2 | Soft agar |
| 3 | Soft agar containing 0.5 mM histidine and biotin |
| 4 | Soft agar containing 5 µg/mL tryptophan |
| 5 | Nutrient agar |
| 6 | Oxoid Nutrient broth No. 2 |
| 7 | PBS (pH 7.4) |

**Chemicals**

| **Chemical** | **Batch No./Lot No.** | **Manufactured by** |
| --- | --- | --- |
| Agar [Difco] | 9057993 | Difco Laboratories  Becton Dickinson and Company, MD, USA |
| Oxoid nutrient Broth No. 2 | 2378115 | Oxoid Ltd., Basingstoke,  Hampshire England |
| Potassium chloride | K16A/1016/0208/61 | S. D. Fine Chem Ltd, Mumbai,  India |
| NADP | 6804 | Chemquest GmbH, Germany. |
| Glucose-6-phosphate | 7366201 | Sisco Research Laboratories Pvt.  Ltd, Maharashtra, India |
| Ammonium sodium  phosphate. 4H2O | MKCH8396 | Sigma Aldrich St. Louis, USA |
| L-histidine | BCBP4059V |  |
| Magnesium Chloride | S5686533720 | Merck KGaA, Darmstadt, Germany |
| Citric acid monohydrate | DB9D690337 | Merck Life Science Private Limited, Mumbai, India. |
| Sodium Chloride | DI7D672243 |  |
| Di-Potassium hydrogen  phosphate anhydrous | DC7D670460 |  |
| Dextrose | DI8P682319 |  |
| Nutrient agar | 0000302104 | Hi Media Laboratories, Private Limited Mumbai India |
| Agar Agar | 0000361577 |  |
| Magnesium sulphate.7H2O | 0000323390 |  |
| Phosphate buffered saline | 0000341565 |  |
| L-Tryptophan | 0000328420 |  |
| D-biotin | 0000413842 |  |
| DMSO | R154M18 | Avantor Performance Materials  India Limited, Maharashtra, India. |

**Preparation of S9 Homogenate and Activation Mixture**

Aroclor 1254 induced rat liver S9 homogenate was used as the metabolic activation system. The S9 homogenate was prepared from male Wistar rats induced with a single intraperitoneal injection of Aroclor 1254 (0.7 mL/rat ready to use solution), 5 days prior to sacrifice. The S9 homogenate was prepared in batches and stored in a deep freezer maintained at -68 to -86ºC.

S9 homogenate was thawed immediately before use and mixed with the cofactor solution containing 4 mM NADP, 5 mM glucose-6-phosphate, 8 mM MgCl2 and 33 mM KCl in PBS.

**S9 Mix**

| **Co-factor** | **Preliminary toxicity test (mg)** | **Mutation assay (mg)** |
| --- | --- | --- |
| NADP (4 mM) | 28.35 | 157.47 |
| Glucose-6-phosphate (5 mM) | 15.31 | 85.03 |
| Magnesium chloride (8 mM) | 14.64 | 81.32 |
| Potassium chloride (33 mM) | 22.14 | 123.01 |

The co-factors solution was prepared by dissolving the following in 9 and 50 mL of cold PBS for the preliminary toxicity test and the mutation assay, respectively. This solution was filter sterilized using a 0.2 µm disposable syringe filter.

S9 mix was prepared by mixing 1 and 5.5 mL of the S9 homogenate with 9 and 49.5 mL of the co-factors solution for the preliminary toxicity test and the mutation assay, respectively, kept in an ice bath and used within one hour.

**Test Item Stock and Dilutions**

| **USKECSE stock or dilution** | **Volume of vehicle (DMSO)** | **USKECSE concentration / mL of vehicle** | **Final**  **USKECSE dose/plate** | **Group No.** |
| --- | --- | --- | --- | --- |
| A. 1 mL stock | 4 mL | 10000 μg | NA | NA |
| B. 0.5 mL A | 9.5 mL | 500 μg | 50 µg | G2 |
| C. 0.79 mL A | 4.21 mL | 1580 μg | 158 µg | G3 |
| D. 2.5 mL A | 2.5 mL | 5000 µg | 500 μg | G4 |
| E. 1.581 mL stock | 3.419 mL | 15810 µg | 1581 µg | G5 |
| F. 100 µL stock | NA | 50000 µg | 5000 µg | G6 |

NA – Not Applicable

**Performance of the Assay**

Mutation assay was carried out using the pre-incubation mode of exposure as follows:

**A. Presence of Metabolic Activation**

a) 100 µL test dose /vehicle/appropriate positive control

b) 100 µL bacterial culture

c) 500 µL S9 mix

**B. Absence of Metabolic Activation**

a) 100 µL test dose/vehicle/appropriate positive control

b) 100 µL bacterial culture

c) 500 µL Phosphate Buffered Saline

These test constituents were transferred into sterile tubes and maintained in an incubator shaker for approximately 20 to 30 minutes at 37 ± 1 ºC. At the end of the incubation period, 2 mL soft agar containing histidine-biotin/tryptophan was added to each of the tubes, mixed and overlaid onto VB agar plates. After the soft agar sets, the plates were incubated at 37 ± 1 °C for 67 hours. Revertant colonies were counted manually, and the bacterial background lawn on each plate was examined for test item toxicity.

**Viable Counts**

The bacterial suspension of each tester strain was diluted up to 10^-6^ dilution in PBS. One hundred microliters (100 µL) from the 10^-6^ dilution of each tester strain was mixed with 2 mL soft agar and plated onto nutrient agar plates in triplicate. The plates were incubated at 37 ± 1°C for 67 hours for the initial as well as the confirmatory mutation assays. After incubation, the number of colonies in each plate were manually counted and expressed as the number of colony forming units per mL of the bacterial suspension.

**Effect on Bacterial Background Lawn**

The condition of the bacterial background lawn was evaluated for evidence of the test item toxicity using the code system, presented in Appendix 3.

**Number of Revertants**

Revertant colonies of all the tester strains for the controls and each test dose were counted manually.

**Viable Counts**

Colonies of all the tester strains on nutrient agar plates were counted manually.

**1.2 In *vitro* mammalian chromosomal aberration test in human peripheral blood lymphocytes**

The clastogenic potential of the kaempferol enriched *Camellia sinensis* extract (USKECSE) to induce chromosomal aberrations in mammalian cells was evaluated using cultured human peripheral blood lymphocytes. This study was conducted in accordance with the OECD Guideline No. 473 for testing of chemicals, “In Vitro Mammalian Chromosomal Aberration Test”, adopted: 29 July 2016 (OECD, 2016).

### Controls

#### Vehicle Control

DMSO was used as the vehicle control

#### Positive Controls

DMSO was used to prepare the stock and dilutions of the following positive controls:

| **Activation** | **Positive controls**  **[CAS No.]** | **Concentration (µg/mL)** | **Batch/Lot No.** | **Manufactured by** |
| --- | --- | --- | --- | --- |
| Presence | Cyclophosphamide monohydrate (CPA)  [6055-19-2) | 14 | MKBS0021V | Sigma Aldrich Co. St. Louis, MO 63103, USA |
| Absence | Ethyl methanesulphonate (EMS)  [62-50-0] | 600 | BCBQ0451V |  |

### Test System and its source

Human blood cultures were established and used as the test system. Blood was collected from an individualmale donor of age 34 years for chromosomal aberration test.

### Test Medium, Solutionsand Other Chemicals

| **Name** | **Lot/Batch No.** | **Manufacturer** |
| --- | --- | --- |
| Colchicine | SLCB8521 | Sigma Aldrich Co.,  St. Louis, MO 63103, USA |
| Amphoterecin | 065M4051V |  |
| Glucose-6-phosphate | 7366201 | Sisco Research Laboratories Private Limited,  Taloja, Maharashtra, India |
| Fetal Bovine Serum | 42A0289K | Life Technologies Limited, Paisley, UK |
| RPMI 1640 medium | 2110285 |  |
| Phytohaemagglutinin | 2090248 |  |
| Methanol | SK8SA1860 | Merck Life Sciences Private Limited, Mumbai-400 079, India |
| NADP | 6804 | ChemquestT GmbH,  2286, Salzburg, Germany |
| DPX Mountant | J17A/1917/1210/73 | SDFCL, Worli Road,  Mumbai-400 030, India |
| Potassium chloride | K16A/1016/0208/31 |  |
| Penicillin | 0000339571 | Hi-Media LaboratoriesPrivateLimited  Mumbai, India |
| Dulbecco’s Phosphate buffered saline (PBS) | 0000364788 |  |
| Sodium bicarbonate | 0000391954 |  |
| Streptomycin | 0000326702 |  |
| DMSO | R322B18 | Avantor Performance Materials India Limited, Thane, Maharashtra-400607, India |
| Acetic acid | G021J18 |  |
| Xylene | T026K17 |  |
| Giemsa’s stain | 258682527JR | FinarLimited,Charcharwadi, vasna, Sanand, Ahmedabad, Gujarat, India |

### Preparation of S9 Homogenate and Activation Mixture

### Aroclor 1254 induced rat liver S9 homogenate was used as the metabolic activation system. The S9 homogenate was prepared from male Wistar rats induced with a single intraperitoneal injection of Aroclor 1254 (0.7 mL/rat ready to use solution), 5 days prior to sacrifice. The S9 homogenate was prepared in batches and stored in a deep freezer maintained at -68 to -86 ºC. Each batch of S9 homogenate was assessed for sterility, protein content (modified Lowry Assay, Sword and Thomson, 1980) and for its ability to metabolize the promutagens 2-Aminoanthracene and Benzo (a) pyrene to mutagens using Salmonella typhimurium TA100 strain.

### The S9 homogenate was thawed immediately before use and mixed with the co-factor solution containing 25 mg/mL NADP, 180 mg/mL Glucose-6-phosphate, 150 mMKCl and S9 fraction (at a ratio of 1:1:1:2 v/v).

#### S9 Mix

#### The co-factor solution was prepared by dissolving each of the following cofactors in 1.25 mL of RPMI 1640 for the chromosomal aberration assay. These individual components are then in 3.75 ml for the chromosomal aberration assay and sterilized by using a 0.2 µm disposable syringe filter.

| **Co-factor** | **Quantity (mg)**  **Chromosomal aberration assay** |
| --- | --- |
| NADP (25 mg/mL) | 31 |
| Glucose-6-phosphate (180 mg/mL) | 225 |
| Potassium chloride (150 mM) | 14 |

S9 mix was prepared by mixing 6 mLand 3.75 mL of S9 homogenate with 3.75 mL of the cofactor solution for the chromosomal aberration assay. The mix was kept in an ice bath and used within one hour of preparation.

### Blood Cultures

Whole blood cultures were established in sterile disposable centrifuge tubes by placing 0.8 mL of heparinized blood into 8.55 mL RPMI FBS20 and 0.15 mL reagent grade PHA. Blood cultures were incubated in a tissue culture rotator inside a CO_2_ incubator with humidified atmosphere of 82 to 84%, 5±0.2% CO_2_at 37±1°C for approximately 48 hours.

**Test Concentrations**

Experiments 1 & 2 (presence and absence of metabolic activation with
3-hour exposure) and Experiment 3 (absence of metabolic activation with 22-hour exposure)

a) 69   b) 208 and c) 625 µg/mL

**Spindle Inhibitor**

Colchicine at 0.2 µg/mL was used as the spindle inhibitor.

#### Preparation of USKECSE Stock and Dilutions

#### Experiments 1 & 2 (presence and absence of metabolic activation with 3-hour

#### exposure) and Experiment 3 (absence of metabolic activation with 22-hour

#### exposure)

| **USKECSE**  **Stock/Dilution** | **Volume of**  **DMSO** | **USKECSE concentration / mL of DMSO** | **Final USKECSE concentration / culture** | **Final USKECSE concentration / mL culture** | **Group** |
| --- | --- | --- | --- | --- | --- |
| 1. 1.0 mL dilution B | 3.0 mL | ~6900μg | 690 µg | 69 µg | G2 |
| 1. 1.0 mL stock | 2.0 mL | ~20800μg | 2080 µg | 208 µg | G3 |
| 1. 0.15 mL stock | - | 62500μg | 6250μg | 625 µg | G4 |

#### Exposure to Treatment

On the day of treatment, all test doses were prepared immediately before use and mixed with the test medium in tubes containing the target blood cultures. For the experiment in the presence of metabolic activation, 0.5 mL S9 mix was added to the respective blood cultures to achieve a final concentration of 2% S9 (v/v) in the test medium. Similarly, for experiments in the absence of metabolic activation, 0.5 mL of 150 mMKCl was added to the respective tubes.

To respective tubes, 100 µL of the vehicle, USKECSE and the positive controls diluted to appropriate concentrations were added and mixed. The target cells were exposed to the controls and three concentrations of the test item as follows:

- For Experiments 1 and 3, the target cells in duplicate were exposed to the vehicle control, positive control and the appropriate concentrations of the test item for 3-hours in the presence and for 22-hours in the absence of metabolic activation, respectively.
- For Experiment 2, the target cells in duplicate were exposed to the vehicle control and the appropriate concentrations of the test item for 3-hours in the absence of metabolic activation.

After the exposure period, the cultures from the 3-hour exposure were centrifuged to stop the treatment and the test solutions were removed. The cell pellet was washed twice with PBS and the tubes were re-fed with fresh RPMI FBS20 and incubated for approximately another 19 hourstill harvest.

#### Collection of Metaphase Cells

Approximately, 2 hours before harvest, Colchicine was added to cultures at a final concentration of 0.2 µg/mL to arrest dividing cells in metaphase.

Each culture from the controls and treatment groups was harvested at approximately 22 hours after the beginning of the treatment and processed separately for the preparation of chromosomes. This post-treatment harvest time was selected to ensure that cells are analyzed in the first division metaphase after initiation of treatment.

Cells were harvested by centrifugation at approximately 1000 rpm for
5 minutes. The supernatant was carefully removed and cells re-suspended inwarm 0.56% KCl for 15 minutes to allow cell swelling to occur. Cells were then washed with consecutive changes of cold methanol:acetic acid fixative
(3:1 v/v). This centrifugation procedure was repeated until clear white pellets were obtained. The cell suspension was kept in fixative in the refrigerator before slides were made.

#### Slide Preparation

Cells were pelleted and re-suspended in a minimal amount of fresh fixative so as to give a milky suspension. Several drops of suspension were transferred on to clean glass slides, flame dried and dried on a slide warmer maintained at approximately 40°C. Five slides per replicate were made and were then marked with the study number, treatment group, activation, experiment number, replicate number and the slide number.

The slides were stained with freshly prepared 5% Giemsa stain in distilled water, rinsed with tap water, air dried, immersed in xylene and mounted with DPX. The slides were then coded using random numbers before evaluation by an individual not involved in the scoring process.

**Microscopic Analysis**

Metaphases of three concentrations of the USKECSE, the vehicle control and the positive control (of Experiments 1 and 3) cultures were scored in each of the three experiments.

The slides were scanned and each metaphase spread was examined under the 100x objective. One hundred fifty metaphases from each replicate culture were analyzed for chromosome aberrations. The number of chromosomes in each spread was counted and those containing 44 to 48 chromosomes were evaluated for aberrations. A total of 300 such metaphases evenly distributed amongst the duplicate cultures were evaluated for each concentration group including controls. The chromosome number was recorded for all cells analyzed and the microscope coordinates were recorded for the aberrant cells. Aberrations were recorded as chromatid / chromosome gaps or breaks and exchange figures.

Since gaps are not considered as true aberration, the results are presented as metaphases with aberrations including gaps and excluding gaps.

Polyploidy and Endoreduplication were not observed in any of the three experiments of the chromosome aberration assay.

The total number of metaphases showing one or more aberrations both including and excluding gaps was calculated from a set of 300 metaphases for each concentration group.

To calculate the mitotic index, slides were randomly observed under microscope, 1000 cells were counted in different fields and the number of metaphases per 1000 cells were recorded.

**Statistical analyses**

The statistical analysis of the experimental data was carried out using validated SYSTAT Statistical package ver.12.0. Data were analysed for proportions of aberrant metaphases in each sample, excluding gaps as aberrations. Pooled data from each test concentration and the positive control were compared with the vehicle control using Fischer exact test. All analysis and comparisons were evaluated at 5% (p<0.05) level.

**1.3 In *vivo* mammalian bone marrow chromosomal aberration test in Swiss abbino mice**

The purpose of this study was to assess the clastogenicity of the test item Kaempferol- enriched extract of Camellia sinensis leaves (USKECSE) when administered to Swiss albino mice. This study was performed in accordance with the OECD Guidelines for the Testing of Chemicals, No. 475 “Mammalian Bone Marrow Chromosomal Aberration Test” adopted on 29 July, 2016.

**Vehicle**: 5% Ethanol + 10 % PEG 400 + 0.5% w/v sodium carboxymethyl cellulose - medium viscosity containing 0.1% v/v Tween 80 in Milli Q water.

**Positive Control**: Cyclophosphamide monohydrate. Quantity of 7.5 mg of cyclophosphamide monohydrate was dissolved in 5 mL of Milli-Q water to get a concentration of 1.5 mg/mL.

The following doses of USKECSE were selected for the study along with concurrent vehicle and positive controls

G1 - Vehicle control- 0 mg/kg/day

G2 - Low dose - 500 mg/kg/day

G3 - Mid dose - 1000 mg/kg/day

G4 - High dose - 2000 mg/kg/day

G5- Positive control - 15 mg/kg

**Experimental Design, Group Allocation and Number of Mice**

| **Group No.** | **Group** | **Colour of cage card** | **Dose (mg/kg/day)** | **Concentration (mg/ml)** | **No. of mice** | **Sex** | **Mice numbers** | |
| --- | --- | --- | --- | --- | --- | --- | --- | --- |
|  |  |  |  |  |  |  | **From** | **To** |
| **G1** | **Vehicle**  **control** | **White** | **0** | **0** | **5** | **M** | **Mf4401** | **Mf4405** |
|  |  |  |  |  | **5** | **F** | **Mf4406** | **Mf4410** |
| **G2** | **Low dose** | **Yellow** | **500** | **50** | **5** | **M** | **Mf4411** | **Mf4415** |
|  |  |  |  |  | **5** | **F** | **Mf4416** | **Mf4420** |
| **G3** | **Mid dose** | **Green** | **1000** | **100** | **5** | **M** | **Mf4421** | **Mf4425** |
|  |  |  |  |  | **5** | **F** | **Mf4426** | **Mf4430** |
| **G4** | **High dose** | **Pink** | **2000** | **200** | **5** | **M** | **Mf4431** | **Mf4435** |
|  |  |  |  |  | **5** | **F** | **Mf4436** | **Mf4440** |
| **G5** | **Positive**  **control@** | **Blue** | **15** | **1.5** | **5** | **M** | **Mf4441** | **Mf4445** |
|  |  |  |  |  | **5** | **F** | **Mf4446** | **Mf4450** |

**@: Cyclophosphamide monohydrate**

**Treatment**

The dose formulations and the vehicle were administered orally by gavage twice at 24 hours interval with the variation of ± 2 hours at a dose volume of 10 mL/kg body weight.

Cyclophosphamide monohydrate, 15 mg/kg body weight was administered to the mice belonging to the positive control group as a single oral gavage at a dose volume of 10 mL/kg body weight.

Approximately 90 minutes before sacrifice, each mouse was injected intraperitoneally with 0.04% of colchicine at 10 mL per kg body weight to arrest cells in metaphase. The femur bone marrow was flushed and slides were prepared from the cell suspension.

For vehicle control groups, vehicle was administered.

**Clinical Signs and Mortality**

Mice were observed for any clinical signs at pre-dose and approximately 1 hours post dose on the days of treatment and once on the day of sacrifice.

**Body Weight**

For vehicle control and treatment groups, the body weight of mice was recorded on days of administration (Days 1 and 2) and day of sacrifice (Day 3).

For positive control group, the body weight of mice was recorded on Day 1 (the day of treatment) and Day 2 (sacrifice day).

**Terminal sacrifice**

Prior to sacrifice, random numbers were generated for blind coding of mice numbers to avoid bias during evaluation. Prior to sacrifice, the mice were treated with a spindle inhibitor (e.g. colchicine) to arrest the cells in metaphase.

All mice in the vehicle control and treatment groups were sacrificed 21 to 24 hours following the second treatment and were subjected to gross pathological observations. The mice in the positive control group were sacrificed 21 to 24 hours after the single treatment and subjected to gross pathological observations.

The mice sacrificed at term were killed by cervical dislocation and the femora from both sides were removed after clearing the musculature. The femur heads were trimmed to expose the marrow canals and the bone marrow was aspirated into a syringe containing 1-2 mL of 0.56% KCl (hypotonic solution) and collected in a centrifuge tube.

**Processing and Slide Preparation**

Cell suspensions collected in centrifuge tubes were incubated at 37 ºC for 15 minutes and centrifuged at 2000 rpm for 5 minutes. The supernatant was discarded and the cell button was dispersed. The cell suspensions were fixed and processed in freshly prepared cold fixative (methanol: acetic acid, 3:1). Finally the cell button with the cold fixative was left in the refrigerator for 60 minutes. The cell suspension was centrifuged at 2400 rpm for 10 minutes and supernatant was discarded. The cell button was re-dispersed in 0.5 mL of cold fixative. The cell suspension was dropped onto a clean chilled slide, flame dried and placed on a slide warmer. The entire cell suspension obtained for each animal was used for preparation of slides. The slide was marked with the study number, animal code number and slide number. Four slides were prepared for each mouse.

**Staining**

Slides were stained with 10% Giemsa stain for 20 minutes, rinsed in tap water, blow dried, immersed in xylene and cover slips mounted with DPX.

**Microscopic Analysis of Metaphases**

The frequency of mitotic divisions (mitotic index) was estimated by counting the number of metaphase plates per 1000 blast cells per animal. Slides were screened for 200 analyzable metaphases per animal and scored for aberrations classified as chromatid or chromosome type of gaps, breaks, acentric fragments, ring chromosomes, multiple chromatid breaks, pulverization, polyploidy and exchange figures. Since gaps are not considered as true aberrations, the results are presented as metaphases with aberrations and metaphases with aberrations including gaps and excluding gaps.

**Data compilation**

All findings observed were recorded and presented in the report. The individual animal data are presented as appendices and the summarized results with statistical analysis are presented as tables.

**Statistical analyses**

Results of statistical analysis were reported in the form of Mean ± SD and sample size. Statistical analysis of data was carried out using licensed copies of SYSTAT Statistical package ver.12.0. All quantitative variables like changes in body weight were tested for normality (Shapiro-Wilk test) and homogeneity (Levene’s test) of within group variance before performing ANOVA. Data was analyzed for proportions of aberrant metaphases in each sample, including and excluding gaps as aberrations. Pooled data from each treatment group and the positive control was compared with the vehicle control using Fisher exact test. All analyses and comparisons were evaluated at 5% (p < 0.05) level and the statistical significance was designated as given below:

* : Significantly different from the vehicle control group

**2 Acute toxicity study**

The objective of this study was to assess the acute toxicity profile of Kaempferol-enriched extract of *Camellia sinensis* leaves (USKECSE) when administered to Sprague-Dawley Rats by single oral gavage.

The study was persormed in compliance with the following:

• OECD Principles of Good Laboratory Practice [C (97)186/Final].

• US FDA Good Laboratory Practice for Nonclinical Laboratory Studies (21 CFR Part 58).

• The mutually agreed Study Plan and the Standard Operating Procedures of the test facility.

### Experimental design, group allocation and number of rats

| **Group No.** | **Group** | **Colour of**  **cage card** | **Dose**  **(mg/kg)** | **Dose volume**  **(mL/kg)** | **No. of**  **Rats** | **Sex** | **Rat Numbers** | |
| --- | --- | --- | --- | --- | --- | --- | --- | --- |
|  |  |  |  |  |  |  | **From** | **To** |
| G1 | Vehicle Control | White | 0 | 10 | 5  5 | M  F | Ry2301  Ry2306 | Ry2305  Ry2310 |
| G2 | Low dose | Yellow | 500 | 10 | 5  5 | M  F | Ry2311  Ry2316 | Ry2315  Ry2320 |
| G3 | Mid dose | Green | 1000 | 10 | 5  5 | M  F | Ry2321  Ry2326 | Ry2325  Ry2330 |
| G4 | Mid Intermediate  dose | Pink | 1500 | 10 | 5  5 | M  F | Ry2331  Ry2336 | Ry2335  Ry2340 |
| G5 | High dose | Pink | 2000 | 10 | 5  5 | M  F | Ry2341  Ry2346 | Ry2345  Ry2350 |

M = Male, F = Female

**Treatment**

The dose formulations was administered by single oral gavage to the rats in USKECSE dose groups. Similarly, vehicle will be administered to rats in vehicle control group. The dose volume administered to each rat will be 10 mL/kg body weight. The dose volume will be adjusted based on the body weight of individual rat measured before dose administration.

Routine cage side observations for clinical signs was done at least once daily throughout the 15 day experimental period. Only on the day of treatment the observation for clinical signs was done at post-dose. Each rat was observed for mortality and morbidity at least twice daily i.e., once in the morning and once in the afternoon.

## Anatomic pathology

### Gross pathology

### All the rats in the study were subjected to detailed necropsy on Day 15 and findings were recorded. All rats to be sacrificed at term were fasted (overnight), weighed, anaesthetized with isoflurane (as per the random numbers generated for the study), exsanguinated and subjected for gross examination.

### STATISTICAL ANALYSES

Data captured using Provantis^TM^: Parameters such as body weight, food consumption (derived data), terminal fasting body weight were analysed using Provantis^TM^ built-in statistical tests.

All analyses and comparisons were evaluated at the 5% (p<0.05) level.

*: Significantly higher/lower than the vehicle control group.

**Results**

**1.1 Ames assay**

**Table 1.** Viable Counts of Tester Strains in the Overnight Culture

| **Tester Strains** | **Viable Counts (x 109 CFU/mL*) Mutation Assay** |
| --- | --- |
| TA98 | 1.57 |
| TA100 | 1.60 |
| TA1535 | 1.57 |
| TA1537 | 1.60 |
| WP2*uvr*A (pKM101) | 1.67 |

* Required Cell count: 1-2x10^9^ Colony Forming Units (CFU)/mL

**Table 2.** Summary results of bacterial reverse mutation assay in presence of metabolic activation

| **Treatment [µg/plate]** | **No. of revertants/plate^a^** | | | | | | | | | | | | | | | |
| --- | --- | --- | --- | --- | --- | --- | --- | --- | --- | --- | --- | --- | --- | --- | --- | --- |
|  | **TA98** | | | **TA100** | | | **TA1535** | | | **TA1537** | | | **WP2*uvr*A (pKM101)** | | | |
|  | **Mean** | **SD** | **Ratio^b^** | **Mean** | **SD** | **Ratio^b^** | **Mean** | **SD** | **Ratio^b^** | **Mean** | **SD** | **Ratio^b^** | **Mean** | **SD** | **Ratio^b^** |  |
| DMSO | 26 | 2 | NA | 93 | 10 | NA | 14 | 2 | NA | 11 | 1 | NA | 144 | 6 | NA |  |
| 50 | 24 | 2 | 0.91 | 96 | 15 | 1.04 | 13 | 2 | 0.98 | 10 | 1 | 0.94 | 140 | 9 | 0.97 |  |
| 158 | 25 | 2 | 0.97 | 84 | 10 | 0.90 | 13 | 1 | 0.98 | 10 | 2 | 0.97 | 137 | 6 | 0.95 |  |
| 500 | 25 | 4 | 0.96 | 82 | 4 | 0.88 | 14 | 2 | 1.00 | 10 | 1 | 0.97 | 140 | 8 | 0.97 |  |
| 1581 | 25 | 2 | 0.95 | 91 | 8 | 0.98 | 13 | 1 | 0.93 | 10 | 2 | 0.91 | 141 | 3 | 0.98 |  |
| 5000 | 25 | 3 | 0.95 | 89 | 3 | 0.95 | 13 | 1 | 0.93 | 10 | 1 | 0.94 | 139 | 6 | 0.97 |  |
| Positive control | 579 ^c^ | 19 ^c^ | 22.28 ^c^ | 872 ^c^ | 18 ^c^ | 9.38 ^c^ | 178 ^c^ | 10 ^c^ | 13.00 ^c^ | 173 ^c^ | 17 ^c^ | 16.22 ^c^ | 572 ^d^ | 22 ^d^ | 3.97 ^d^ |  |
| ^a^ Values are means of three replicates calculated from individual values of [Appendix 1](#_bookmark69) and are rounded off to the nearest whole number  ^b^ Ratio of treated/Vehicle control (mean revertants per plate). The presentation was made using the mean values with decimals before rounding off to the nearest whole number. Hence, some of the values may not match if calculated using the rounded-off mean values of this summary table.  ^c^ TA98, TA100, TA1535, TA1537: 2-Aminoanthracene (4 µg/plate)  ^d^ WP2*uvrA* (pKM101): 2-Aminoanthracene (30 µg/plate) SD: Standard deviation NA: Not applicable | | | | | | | | | | | | | | | | |

**Table 3.** Summary results of bacterial reverse mutation assay in absence of metabolic activation

| **Treatment [µg/plate]** | **No. of revertants/plate^a^** | | | | | | | | | | | | | | | |
| --- | --- | --- | --- | --- | --- | --- | --- | --- | --- | --- | --- | --- | --- | --- | --- | --- |
|  | **TA98** | | | **TA100** | | | **TA1535** | | | **TA1537** | | | **WP2*uvr*A (pKM101)** | | | |
|  | **Mean** | **SD** | **Ratio^b^** | **Mean** | **SD** | **Ratio^b^** | **Mean** | **SD** | **Ratio^b^** | **Mean** | **SD** | **Ratio^b^** | **Mean** | **SD** | **Ratio^b^** |  |
| DMSO | 26 | 3 | NA | 91 | 4 | NA | 14 | 1 | NA | 10 | 1 | NA | 141 | 3 | NA |  |
| 50 | 25 | 3 | 0.96 | 91 | 9 | 0.99 | 13 | 2 | 0.98 | 9 | 1 | 0.90 | 140 | 6 | 0.99 |  |
| 158 | 26 | 2 | 0.99 | 86 | 9 | 0.94 | 12 | 1 | 0.90 | 10 | 2 | 0.97 | 138 | 9 | 0.98 |  |
| 500 | 24 | 1 | 0.92 | 88 | 10 | 0.97 | 13 | 1 | 0.95 | 10 | 1 | 0.97 | 144 | 4 | 1.02 |  |
| 1581 | 25 | 3 | 0.97 | 92 | 11 | 1.01 | 13 | 1 | 0.93 | 10 | 1 | 0.94 | 139 | 10 | 0.98 |  |
| 5000 | 25 | 2 | 0.97 | 88 | 8 | 0.96 | 13 | 2 | 0.95 | 10 | 2 | 0.94 | 140 | 7 | 0.99 |  |
| Positive control | 268 ^c^ | 24 ^c^ | 10.29 ^c^ | 588 ^d^ | 9 d | 6.43 ^d^ | 176 ^d^ | 20 ^d^ | 12.90 ^d^ | 178 ^e^ | 19 ^e^ | 17.26 ^e^ | 578 ^f^ | 12 ^f^ | 4.09 ^f^ |  |
| ^a^ Values are means of three replicates calculated from individual values of [Appendix 2](#_bookmark70) and are rounded off to the nearest whole number  ^b^ Ratio of treated/Vehicle control (mean revertants per plate). The presentation was made using the mean values with decimals before rounding off to the nearest whole number. Hence, some of the values may not match if calculated using the rounded-off mean values of this summary table.  ^c^ TA98: 2-Nitrofluorene (2 µg/plate),  ^d^ TA100, TA1535: Sodium azide (1 µg/plate) ^e^ TA1537: 9-Aminoacridine (50 µg/plate)  ^f^ WP2*uvrA* (pKM101): 4-Nitroquinoline-1-oxide (4 µg/plate) SD: Standard deviation NA: Not applicable | | | | | | | | | | | | | | | | |

| **Treatment**  **(µg/mL)** | **No. of metaphases scored** | **No.(%) of metaphases with aberrations** | | | | | | | **Total No.(%) of aberrant metaphases^*^** | | **Relative Mitotic**  **Inhibition**  **(%)** |
| --- | --- | --- | --- | --- | --- | --- | --- | --- | --- | --- | --- |
|  |  | **Gaps** | | **Breaks** | | **Exchanges** | | **Ring** | **Including**  **Gaps** | **Excluding**  **Gaps** |  |
|  |  | **Cs** | **Ct** | **Cs** | **Ct** | **Cs** | **Ct** |  |  |  |  |
| DMSO  (100 µL) | 300 | 0 | 0 | 0 | 1  (0.33) | 0 | 0 | 0 | 1  (0.33) | 1  (0.33) | 0 |
| 69 | 300 | 0 | 0 | 0 | 0 | 0 | 0 | 0 | 0 | 0 | 28 |
| 208 | 300 | 0 | 0 | 0 | 1  (0.33) | 0 | 0 | 0 | 1  (0.33) | 1  (0.33) | 37 |
| 625 | 300 | 0 | 0 | 0 | 1  (0.33) | 0 | 0 | 0 | 1  (0.33) | 1  (0.33) | 45 |
| CPA 14 | 300 | 5  (1.67) | 3  (1.0) | 10  (3.33) | 47  (15.67) | 19  (6.33) | 37  (12.33) | 1  (0.33) | 98  (32.67) | 98  (32.67) | 48 |

**1.2 In *vitro* mammalian chromosomal aberration test in human peripheral blood lymphocytes**

**TABLE 4.** Summary Results of Chromosomal Aberration Test - Experiment 1

Refer appendix [4](#App5)

*: Metaphase plate with one or more than one aberrations considered as one metaphase plate with aberrations

Cs: Chromosome type Ct: Chromatid type CPA: Cyclophosphamide monohydrate RC: Ring Chromosome

+: Significantly higher than vehicle control (p < 0.05) by Fischer exact test

Note: There were no incidences of polyploidy and endoreduplicated cells

**TABLE 5.** Summary Results of Chromosomal Aberration Test - Experiment 2

| **Treatment**  **(µg/mL)** | **No. of metaphases scored** | **No.(%) of metaphases with aberrations** | | | | | | **Total No.(%) of aberrant metaphases^*^** | | **Mitotic**  **Inhibition**  **(%)** |
| --- | --- | --- | --- | --- | --- | --- | --- | --- | --- | --- |
|  |  | **Gaps** | | **Breaks** | | **Exchanges** | | **Including**  **Gaps** | **Excluding**  **Gaps** |  |
|  |  | **Cs** | **Ct** | **Cs** | **Ct** | **Cs** | **Ct** |  |  |  |
| DMSO  (100 µL) | 300 | 0 | 0 | 0 | 0 | 0 | 0 | 0 | 0 | 0 |
| 69 | 300 | 0 | 0 | 0 | 0 | 0 | 0 | 0 | 0 | 26 |
| 208 | 300 | 0 | 0 | 0 | 0 | 0 | 0 | 0 | 0 | 34 |
| 625 | 300 | 0 | 0 | 0 | 0 | 0 | 0 | 0 | 0 | 42 |

Refer appendix [5](#App5)

*: Metaphase plate with one or more than one aberrations considered as one metaphase plate with aberrations

Cs: Chromosome type Ct: Chromatid type

Note: There were no incidences of polyploidy and endoreduplicated cells

**TABLE 6.** Summary Results of Chromosomal Aberration Test - Experiment 3

Refer appendix [6](#App6)

| **Treatment**  **(µg/mL)** | **No. of metaphases scored** | **No.(%) of metaphases with aberrations** | | | | | | | **Total No.(%) of aberrant metaphases^*^** | | **Mitotic**  **Inhibition**  **(%)** |
| --- | --- | --- | --- | --- | --- | --- | --- | --- | --- | --- | --- |
|  |  | **Gaps** | | **Breaks** | | **Exchanges** | | **Ring** | **Including**  **Gaps** | **Excluding**  **Gaps** |  |
|  |  | **Cs** | **Ct** | **Cs** | **Ct** | **Cs** | **Ct** |  |  |  |  |
| DMSO  (100 µL) | 300 | 0 | 0 | 0 | 1  (0.33) | 0 | 0 | 0 | 1  (0.33) | 1  (0.33) | 0 |
| 69 | 300 | 0 | 0 | 0 | 0 | 0 | 0 | 0 | 0 | 0 | 30 |
| 208 | 300 | 0 | 0 | 0 | 0 | 0 | 0 | 0 | 0 | 0 | 39 |
| 625 | 300 | 0 | 0 | 0 | 1  (0.33) | 0 | 0 | 0 | 1  (0.33) | 1  (0.33) | 48 |
| EMS 600 | 300 | 4  (1.33) | 1  (0.33) | 22  (7.33) | 31  (10.33) | 21  (7.00) | 30  (10.00) | 1  (0.33) | 94  (31.33) | 93  (31.00) | 49 |

*: Metaphase plate with one or more than one aberrations considered as one metaphase plate with aberrations Cs: Chromosome type Ct: Chromatid type RC: Ring Chromosome EMS: Ethyl methanesulfonate

+: Significantly higher than vehicle control (p < 0.05) by Fischer exact test

Note: There were no incidences of polyploidy and endoreduplicated cells

**1.3 In *vivo* mammalian bone marrow chromosomal aberration test in Swiss albino mice**

**TABLE 7.** Details of Experimental Design, Treatment and Sacrifice Schedule

| **Group** | **Dose**  **(mg/kg/day)** | **Dosage**  **volume**  **(mL/kg)** | **Dose groups** | **No. of mice per group** | | | **Treatment and sacrifice schedule** |
| --- | --- | --- | --- | --- | --- | --- | --- |
|  |  |  |  | **M** | **F** | **Total** |  |
| **G1** | **0** | **10** | **Vehicle Control #** | **5** | **5** | **10** | **Mice in the vehicle/test item groups were administered vehicle/test item by oral gavage twice at an interval of 24 hours and sacrificed 21-24 hours after the second dose. The mice in the positive control group were treated with Cyclophosphamide**  **monohydrate by a single oral gavage and sacrificed 21-24 hours after dosing. Approximately 90 minutes before sacrifice, each mouse was injected intraperitoneally with 0.04% of colchicine at 10 mL per kg body weight to arrest cells in metaphase.** |
| **G2** | **500** | **10** | **Low Dose** | **5** | **5** | **10** |  |
| **G3** | **1000** | **10** | **Mid dose** | **5** | **5** | **10** |  |
| **G4** | **2000** | **10** | **High Dose** | **5** | **5** | **10** |  |
| **G5** | **15** | **10** | **Positive Control @** | **5** | **5** | **10** |  |

#: 5% Ethanol + 10 % PEG 400 + 0.5% w/v sodium carboxymethyl cellulose - medium viscosity containing 0.1% v/v Tween 80 in Milli Q water

@ : Cyclophosphamide monohydrate

**TABLE 8.** Summary of Body Weights, Clinical Signs, Mortality and Necropsy Findings

Refer Appendix 7

**Male**

| Group &  Dose  (mg/kg/day) | No. of  Mice | Body weight (g) on | | | Bwt Change (g)  (Day 3- Day 1) | Bwt Change (g)  (Day 2- Day 1) | Clinical signs | Mortality | Necropsy findings |
| --- | --- | --- | --- | --- | --- | --- | --- | --- | --- |
|  |  | Day 1 | Day 2 | Day 3 |  |  |  |  |  |
| G1  0 | 5 | 35.24  2.49 | 35.18  2.59 | 36.05  2.61 | 0.80  0.82 | -0.06  0.42 | NAD | None | NAD |
| G2  500 | 5 | 35.12  2.77 | 34.87  3.01 | 35.38  3.41 | 0.25  0.86 | ---  --- | NAD | None | NAD |
| G3  1000 | 5 | 34.84  2.85 | 34.05  2.46 | 34.04  3.30 | -0.80  0.96 | ---  --- | NAD | None | NAD |
| G4  2000 | 5 | 35.07  3.22 | 34.71  3.38 | 35.42  2.86 | 0.35  0.93 | ---  --- | NAD | None | NAD |
| G5@  15 | 5 | 35.73  3.55 | 36.23  3.44 | ---  --- | ---  --- | 0.50*  0.33 | NAD | None | NAD |

@: Positive control (Cyclophosphamide monohydrate) NAD: No Abnormality Detected Bwt: Body weight

Values: Mean ± SD

*: Significantly different from the vehicle control group at p<0.05

**TABLE 8** contd. Summary of Body Weights, Clinical Signs, Mortality and Necropsy Findings

Refer Appendix 7

**Female**

| Group &  Dose  (mg/kg/day) | No. of  Mice | Body weight (g) on | | | Bwt Change (g)  (Day 3- Day 1) | Bwt Change (g)  (Day 2- Day 1) | Clinical signs | Mortality | Necropsy findings |
| --- | --- | --- | --- | --- | --- | --- | --- | --- | --- |
|  |  | Day 1 | Day 2 | Day 3 |  |  |  |  |  |
| G1  0 | 5 | 28.26  1.18 | 28.08  1.42 | 28.81  1.35 | 0.55  0.18 | -0.18  0.45 | NAD | None | NAD |
| G2  500 | 5 | 28.45  1.57 | 28.11  1.73 | 29.06  2.26 | 0.61  1.19 | ---  --- | NAD | None | NAD |
| G3  1000 | 5 | 27.67  1.32 | 27.25  1.82 | 27.88  1.88 | 0.21  0.84 | ---  --- | NAD | None | NAD |
| G4  2000 | 5 | 28.52  1.46 | 29.20  1.76 | 30.69  1.91 | 2.17*  1.02 | ---  --- | NAD | None | NAD |
| G5@  15 | 5 | 29.63  1.74 | 30.09  1.35 | ---  --- | ---  --- | 0.46  0.62 | NAD | None | NAD |

@: Positive control (Cyclophosphamide monohydrate) NAD: No Abnormality Detected Bwt: Body weight

Values: Mean ± SD

*: Significantly different from the vehicle control group at p<0.05

**TABLE 9.** Summary of Chromosomal Aberrations Analysis and Mitotic Index

Refer Appendix 8

( ): Percentage values

#: Metaphase plate with one or more aberrations considered as one metaphase plate with aberrations. Mitotic index: No. of metaphase plates per 1000 blast cells

*: Significantly different from vehicle control group at p<0.05

MP: Metaphase Plate Ct: Chromatid Cs: Chromosome BC: Blast cell

**TABLE 9** contd. Summary of Chromosomal Aberrations Analysis and Mitotic Index

Refer Appendix 8

( ): Percentage values

#: Metaphase plate with one or more aberrations considered as one metaphase plate with aberrations. Mitotic index: No. of metaphase plates per 1000 blast cells

*: Significantly different from vehicle control group at p<0.05

MP: Metaphase Plate Ct: Chromatid Cs: Chromosome BC: Blast cell

**TABLE 9** contd. Summary of Chromosomal Aberrations Analysis and Mitotic Index

Refer Appendix 8

( ): Percentage values

#: Metaphase plate with one or more aberrations considered as one metaphase plate with aberrations. Mitotic index: No. of metaphase plates per 1000 blast cells

*: Significantly different from vehicle control group at p<0.05

MP: Metaphase Plate Ct: Chromatid Cs: Chromosome BC: Blast cell

**APPENDICES**

**1.1 Ames assay**

**APPENDIX 1.** Results of Bacterial Reverse Mutation Assay in the Presence of Metabolic Activation – Individual Plate Counts

| **Treatment [µg/plate]** | **No. of revertants / plate** | | | | | **Bacterial lawn* intensity** | **Precipitation*** |
| --- | --- | --- | --- | --- | --- | --- | --- |
|  | **TA 98** | **TA 100** | **TA 1535** | **TA 1537** | **WP2*uvr*A (pKM101)** |  |  |
| DMSO | 24 | 95 | 14 | 10 | 146 | 4+ | NPO |
|  | 26 | 102 | 12 | 12 | 149 |  |  |
|  | 28 | 82 | 15 | 10 | 138 |  |  |
| 50 | 22 | 80 | 15 | 11 | 130 | 4+ | NPO |
|  | 25 | 99 | 12 | 10 | 142 |  |  |
|  | 24 | 110 | 13 | 9 | 148 |  |  |
| 158 | 23 | 91 | 13 | 9 | 141 | 4+ | NPO |
|  | 26 | 72 | 13 | 12 | 139 |  |  |
|  | 27 | 88 | 14 | 10 | 130 |  |  |
| 500 | 29 | 79 | 15 | 11 | 140 | 4+ | NPO |
|  | 24 | 86 | 14 | 10 | 132 |  |  |
|  | 22 | 80 | 12 | 10 | 147 |  |  |
| 1581 | 23 | 82 | 12 | 9 | 145 | 4+ | NPO |
|  | 26 | 94 | 13 | 12 | 140 |  |  |
|  | 25 | 97 | 13 | 8 | 139 |  |  |
| 5000 | 24 | 91 | 13 | 9 | 133 | 4+ | NPO |
|  | 22 | 86 | 12 | 11 | 145 |  |  |
|  | 28 | 89 | 13 | 10 | 140 |  |  |
| Positive control ^a,^ ^b^ | 560 | 870 | 188 | 158 | 549 | 4+ | NPO |
|  | 598 | 891 | 176 | 169 | 576 |  |  |
|  | 580 | 856 | 169 | 192 | 592 |  |  |
| *Refer Appendix 3  ^a^ TA98, TA100, TA1535, TA1537: 2-Aminoanthracene (4 µg/plate)  ^b^ WP2*uvrA* (pKM101): 2-Aminoanthracene (30 µg/plate) | | | | | | | |

**APPENDIX 2.** Results of Bacterial Reverse Mutation Assay in the Absence of Metabolic Activation – Individual Plate Counts

| **Treatment [µg/plate]** | **No. of revertants / plate** | | | | | **Bacterial lawn* intensity** | **Precipitation*** |
| --- | --- | --- | --- | --- | --- | --- | --- |
|  | **TA 98** | **TA 100** | **TA 1535** | **TA 1537** | **WP2*uvr*A (pKM101)** |  |  |
| DMSO | 23 | 88 | 13 | 11 | 145 | 4+ | NPO |
|  | 29 | 96 | 14 | 10 | 139 |  |  |
|  | 26 | 90 | 14 | 10 | 140 |  |  |
| 50 | 24 | 82 | 15 | 10 | 142 | 4+ | NPO |
|  | 23 | 99 | 12 | 9 | 144 |  |  |
|  | 28 | 91 | 13 | 9 | 133 |  |  |
| 158 | 27 | 94 | 12 | 12 | 136 | 4+ | NPO |
|  | 26 | 87 | 12 | 10 | 130 |  |  |
|  | 24 | 77 | 13 | 8 | 148 |  |  |
| 500 | 25 | 78 | 14 | 9 | 143 | 4+ | NPO |
|  | 23 | 89 | 12 | 11 | 148 |  |  |
|  | 24 | 98 | 13 | 10 | 140 |  |  |
| 1581 | 26 | 95 | 13 | 10 | 130 | 4+ | NPO |
|  | 22 | 102 | 12 | 10 | 149 |  |  |
|  | 28 | 80 | 13 | 9 | 137 |  |  |
| 5000 | 25 | 86 | 12 | 8 | 133 | 4+ | NPO |
|  | 27 | 81 | 12 | 12 | 147 |  |  |
|  | 24 | 97 | 15 | 9 | 141 |  |  |
| Positive control a, b, c, d | 291 | 596 | 160 | 160 | 567 | 4+ | NPO |
|  | 268 | 579 | 198 | 198 | 590 |  |  |
|  | 244 | 588 | 171 | 177 | 578 |  |  |
| *Refer Appendix 3  ^a^ TA98: 2-Nitrofluorene (2 µg/plate),  ^b^ TA100, TA1535: Sodium azide (1 µg/plate)  ^c^ TA1537: 9-Aminoacridine (50 µg/plate)  ^d^ WP2*uvrA* (pKM101): 4-Nitroquinoline-1-oxide (4 µg/plate) | | | | | | | |

**APPENDIX 3.** In-house Bacterial Background Lawn Evaluation Codes

| **Code** | **Description** | **Characteristics** |
| --- | --- | --- |
| 0 | Absent | Distinguished by a complete lack of any micro-colony lawn over > 90% of the plate compared with the vehicle control plates |
| 1+ | Severely Reduced | Distinguished by an extreme thinning of the micro- colony lawn resulting in an increase in the size of the micro-colonies compared with the vehicle control plates such that the micro-colony lawn is visible to the unaided eye as isolated colonies |
| 2+ | Moderately reduced | Distinguished by a marked thinning of the micro-colony lawn resulting in a pronounced increase in the size of the micro-colonies compared with the vehicle control plates |
| 3+ | Slightly reduced | Distinguished by a noticeable thinning of the micro- colony lawn and possibly a slight increase in the size of the micro-colonies compared with the vehicle control plates |
| 4+ | Normal | Distinguished by a healthy background lawn comparable to vehicle control plates |
| NP | Non- interfering precipitate | Distinguished by precipitate on the plate that is visible to the naked eye and is not interfering with the evaluation of bacterial lawn |
| IP | Interfering precipitate | Distinguished by precipitate on the plate that is visible to the naked eye and is interfering with the evaluation of bacterial lawn |
| OP | Obscured by particulate | The background lawn cannot be accurately evaluated due to microscopic test item particulate |
| NPO | No precipitation  observed | Precipitation not observed |

**1.2 In *vitro* mammalian chromosomal aberration test in human peripheral blood lymphocytes**

| Group No. | Treatment (µg/mL) | **Repli-cate**  **No.** | **No. of meta-phases scored** | **Normal meta-phases** | **Aberrant metaphases*** | | | | | | | | | |
| --- | --- | --- | --- | --- | --- | --- | --- | --- | --- | --- | --- | --- | --- | --- |
|  |  |  |  |  | **Total No. of Aberrant Metaphases** | | **Chromosome** | | | **Chromatid** | | | **Ring** | |
|  |  |  |  |  | **Including Gaps** | **Excluding Gaps** | **Gaps** | **Breaks** | **Excha-nges** | **Gaps** | **Breaks** | **Excha-nges** |  |  |
| G1 | DMSO (100 µL) | 1 | 150 | 149 | 1 | 1 | 0 | 0 | 0 | 0 | 1 | 0 | 0 |  |
|  |  | 2 | 150 | 150 | 0 | 0 | 0 | 0 | 0 | 0 | 0 | 0 | 0 |  |
| G2 | 69 | 1 | 150 | 150 | 0 | 0 | 0 | 0 | 0 | 0 | 0 | 0 | 0 |  |
|  |  | 2 | 150 | 150 | 0 | 0 | 0 | 0 | 0 | 0 | 0 | 0 | 0 |  |
| G3 | 208 | 1 | 150 | 149 | 1 | 1 | 0 | 0 | 0 | 0 | 1 | 0 | 0 |  |
|  |  | 2 | 150 | 150 | 0 | 0 | 0 | 0 | 0 | 0 | 0 | 0 | 0 |  |
| G4 | 625 | 1 | 150 | 150 | 0 | 0 | 0 | 0 | 0 | 0 | 0 | 0 | 0 |  |
|  |  | 2 | 150 | 149 | 1 | 1 | 0 | 0 | 0 | 0 | 1 | 0 | 0 |  |
| G5 | CPA 14 | 1 | 150 | 100 | 50 | 50 | 1 | 1 | 10 | 1 | 23 | 21 | 0 |  |
|  |  | 2 | 150 | 102 | 48 | 48 | 4 | 9 | 9 | 2 | 24 | 16 | 1 |  |

**APPENDIX 4**. Aberrant Metaphases found in Experiment 1

* Metaphase plate with one or more than one aberrations considered as one metaphase plate with aberrations

CPA: Cyclophosphamide monohydrate

| Group No. | Treatment (µg/mL) | **Repli-cate**  **No.** | **No. of meta-phases scored** | **Normal meta-phases** | **Aberrant metaphases*** | | | | | | | |
| --- | --- | --- | --- | --- | --- | --- | --- | --- | --- | --- | --- | --- |
|  |  |  |  |  | **Total No. of Aberrant Metaphases** | | **Chromosome** | | | **Chromatid** | | |
|  |  |  |  |  | **Including Gaps** | **Excluding Gaps** | **Gaps** | **Breaks** | **Excha-nges** | **Gaps** | **Breaks** | **Excha-nges** |
| G1 | DMSO (100 µL) | 1 | 150 | 150 | 0 | 0 | 0 | 0 | 0 | 0 | 0 | 0 |
|  |  | 2 | 150 | 150 | 0 | 0 | 0 | 0 | 0 | 0 | 0 | 0 |
| G2 | 69 | 1 | 150 | 150 | 0 | 0 | 0 | 0 | 0 | 0 | 0 | 0 |
|  |  | 2 | 150 | 150 | 0 | 0 | 0 | 0 | 0 | 0 | 0 | 0 |
| G3 | 208 | 1 | 150 | 150 | 0 | 0 | 0 | 0 | 0 | 0 | 0 | 0 |
|  |  | 2 | 150 | 150 | 0 | 0 | 0 | 0 | 0 | 0 | 0 | 0 |
| G4 | 625 | 1 | 150 | 150 | 0 | 0 | 0 | 0 | 0 | 0 | 0 | 0 |
|  |  | 2 | 150 | 150 | 0 | 0 | 0 | 0 | 0 | 0 | 0 | 0 |

**APPENDIX 5**. Aberrant metaphases found in Experiment 2

* Metaphase plate with one or more than one aberrations considere0d as one metaphase plate with aberrations

**APPENDIX 6.** Aberrant metaphases found in Experiment 3

| Group No. | Treatment (µg/mL) | **Repli-cate**  **No.** | **No. of meta-phases scored** | **Normal meta-phases** | **Aberrant metaphases*** | | | | | | | | |
| --- | --- | --- | --- | --- | --- | --- | --- | --- | --- | --- | --- | --- | --- |
|  |  |  |  |  | **Total No. of Aberrant Metaphases** | | **Chromosome** | | | **Chromatid** | | | **Ring** |
|  |  |  |  |  | **Including Gaps** | **Excluding Gaps** | **Gaps** | **Breaks** | **Excha-nges** | **Gaps** | **Breaks** | **Excha-nges** |  |
| G1 | DMSO (100 µL) | 1 | 150 | 149 | 1 | 1 | 0 | 0 | 0 | 0 | 1 | 0 | 0 |
|  |  | 2 | 150 | 150 | 0 | 0 | 0 | 0 | 0 | 0 | 0 | 0 | 0 |
| G2 | 69 | 1 | 150 | 150 | 0 | 0 | 0 | 0 | 0 | 0 | 0 | 0 | 0 |
|  |  | 2 | 150 | 150 | 0 | 0 | 0 | 0 | 0 | 0 | 0 | 0 | 0 |
| G3 | 208 | 1 | 150 | 150 | 0 | 0 | 0 | 0 | 0 | 0 | 0 | 0 | 0 |
|  |  | 2 | 150 | 150 | 0 | 0 | 0 | 0 | 0 | 0 | 0 | 0 | 0 |
| G4 | 625 | 1 | 150 | 149 | 1 | 1 | 0 | 0 | 0 | 0 | 1 | 0 | 0 |
|  |  | 2 | 150 | 150 | 0 | 0 | 0 | 0 | 0 | 0 | 0 | 0 | 0 |
| G5 | EMS 600 | 1 | 150 | 105 | 45 | 45 | 2 | 6 | 11 | 0 | 20 | 18 | 0 |
|  |  | 2 | 150 | 101 | 49 | 48 | 2 | 16 | 10 | 1 | 11 | 12 | 1 |

* Metaphase plate with one or more than one aberrations considered as one metaphase plate with aberrations

EMS: Ethyl methanesulfonate

**1.2 In *vitro* mammalian chromosomal aberration test in human peripheral blood lymphocytes**

**APPENDIX 7.** Individual Body Weight, Clinical Signs and Necropsy Findings

| **Group & Dose (mg/kg/day)** | **Mice No.** | **Sex** | **Body weight (g)** | | | **Bwt Change (g) [Day 3 - Day 1]** | **Bwt Change (g) [Day 2 - Day 1]** | **Clinical Signs** | **Necropsy**  **findings** |
| --- | --- | --- | --- | --- | --- | --- | --- | --- | --- |
|  |  |  | **Day 1** | **Day 2** | **Day 3** |  |  |  |  |
| G1 | Mf4401 | M | 31.43 | 31.12 | 32.34 | 0.91 | -0.31 | NAD | NAD |
| 0 | Mf4402 | M | 34.76 | 35.21 | 35.71 | 0.95 | 0.45 | NAD | NAD |
|  | Mf4403 | M | 35.19 | 34.72 | 35.16 | -0.03 | -0.47 | NAD | NAD |
|  | Mf4404 | M | 36.93 | 37.26 | 38.97 | 2.04 | 0.33 | NAD | NAD |
|  | Mf4405 | M | 37.91 | 37.61 | 38.05 | 0.14 | -0.30 | NAD | NAD |
|  | Mf4406 | F | 26.71 | 26.71 | 26.99 | 0.28 | 0.00 | NAD | NAD |
|  | Mf4407 | F | 28.07 | 27.20 | 28.56 | 0.49 | -0.87 | NAD | NAD |
|  | Mf4408 | F | 27.76 | 27.43 | 28.32 | 0.56 | -0.33 | NAD | NAD |
|  | Mf4409 | F | 28.94 | 28.89 | 29.61 | 0.67 | -0.05 | NAD | NAD |
|  | Mf4410 | F | 29.82 | 30.16 | 30.56 | 0.74 | 0.34 | NAD | NAD |
| G2 | Mf4411 | M | 31.08 | 30.97 | 30.86 | -0.22 | --- | NAD | NAD |
| 500 | Mf4412 | M | 34.06 | 33.46 | 34.12 | 0.06 | --- | NAD | NAD |
|  | Mf4413 | M | 35.11 | 34.14 | 34.47 | -0.64 | --- | NAD | NAD |
|  | Mf4414 | M | 37.91 | 38.34 | 39.53 | 1.62 | --- | NAD | NAD |
|  | Mf4415 | M | 37.46 | 37.42 | 37.90 | 0.44 | --- | NAD | NAD |
|  | Mf4416 | F | 26.61 | 26.04 | 28.09 | 1.48 | --- | NAD | NAD |
|  | Mf4417 | F | 27.33 | 27.22 | 27.12 | -0.21 | --- | NAD | NAD |
|  | Mf4418 | F | 28.19 | 27.49 | 27.13 | -1.06 | --- | NAD | NAD |
|  | Mf4419 | F | 29.96 | 29.64 | 31.11 | 1.15 | --- | NAD | NAD |
|  | Mf4420 | F | 30.16 | 30.16 | 31.84 | 1.68 | --- | NAD | NAD |

M: Male F: Female NAD: No Abnormality Detected Bwt: Body weight

**APPENDIX 7.** contd. Individual Body Weight, Clinical Signs and Necropsy Findings

| **Group & Dose (mg/kg/day)** | **Mice No.** | **Sex** | **Body weight (g)** | | | **Bwt Change (g) [Day 3 - Day 1]** | **Bwt Change (g) [Day 2 - Day 1]** | **Clinical Signs** | **Necropsy findings** |
| --- | --- | --- | --- | --- | --- | --- | --- | --- | --- |
|  |  |  | **Day 1** | **Day 2** | **Day 3** |  |  |  |  |
| G3 | Mf4421 | M | 32.06 | 31.30 | 29.99 | -2.07 | --- | NAD | NAD |
| 1000 | Mf4422 | M | 32.24 | 31.96 | 32.40 | 0.16 | --- | NAD | NAD |
|  | Mf4423 | M | 34.61 | 34.64 | 33.71 | -0.90 | --- | NAD | NAD |
|  | Mf4424 | M | 36.64 | 34.94 | 35.30 | -1.34 | --- | NAD | NAD |
|  | Mf4425 | M | 38.67 | 37.39 | 38.81 | 0.14 | --- | NAD | NAD |
|  | Mf4426 | F | 26.73 | 26.83 | 27.74 | 1.01 | --- | NAD | NAD |
|  | Mf4427 | F | 26.01 | 24.31 | 25.14 | -0.87 | --- | NAD | NAD |
|  | Mf4428 | F | 27.74 | 27.71 | 27.26 | -0.48 | --- | NAD | NAD |
|  | Mf4429 | F | 29.16 | 28.79 | 29.72 | 0.56 | --- | NAD | NAD |
|  | Mf4430 | F | 28.73 | 28.62 | 29.56 | 0.83 | --- | NAD | NAD |
| G4 2000 | Mf4431 | M | 31.96 | 31.18 | 32.23 | 0.27 | --- | NAD | NAD |
|  | Mf4432 | M | 31.69 | 31.86 | 33.36 | 1.67 | --- | NAD | NAD |
|  | Mf4433 | M | 36.11 | 35.18 | 35.18 | -0.93 | --- | NAD | NAD |
|  | Mf4434 | M | 36.34 | 35.71 | 36.92 | 0.58 | --- | NAD | NAD |
|  | Mf4435 | M | 39.26 | 39.61 | 39.41 | 0.15 | --- | NAD | NAD |
|  | Mf4436 | F | 26.74 | 27.21 | 29.63 | 2.89 | --- | NAD | NAD |
|  | Mf4437 | F | 27.64 | 28.82 | 29.57 | 1.93 | --- | NAD | NAD |
|  | Mf4438 | F | 28.71 | 29.26 | 29.36 | 0.65 | --- | NAD | NAD |
|  | Mf4439 | F | 28.91 | 28.67 | 30.98 | 2.07 | --- | NAD | NAD |
|  | Mf4440 | F | 30.61 | 32.04 | 33.92 | 3.31 | --- | NAD | NAD |

M: Male F: Female NAD: No Abnormality Detected Bwt: Body weight

**APPENDIX 7.** contd. Individual Body Weight, Clinical Signs and Necropsy Findings

| **Group & Dose (mg/kg)** | **Mice No.** | **Sex** | **Body weight (g)** | | **Bwt Change (g) [Day 2 - Day 1]** | **Clinical Signs** | **Necropsy findings** |
| --- | --- | --- | --- | --- | --- | --- | --- |
|  |  |  | **Day 1** | **Day 2** |  |  |  |
| G5@ | Mf4441 | M | 31.98 | 32.53 | 0.55 | NAD | NAD |
| 15 | Mf4442 | M | 32.79 | 33.18 | 0.39 | NAD | NAD |
|  | Mf4443 | M | 36.36 | 36.92 | 0.56 | NAD | NAD |
|  | Mf4444 | M | 36.66 | 37.62 | 0.96 | NAD | NAD |
|  | Mf4445 | M | 40.86 | 40.92 | 0.06 | NAD | NAD |
|  | Mf4446 | F | 27.39 | 28.92 | 1.53 | NAD | NAD |
|  | Mf4447 | F | 28.58 | 29.00 | 0.42 | NAD | NAD |
|  | Mf4448 | F | 29.87 | 29.97 | 0.10 | NAD | NAD |
|  | Mf4449 | F | 30.32 | 30.28 | -0.04 | NAD | NAD |
|  | Mf4450 | F | 31.97 | 32.26 | 0.29 | NAD | NAD |

@: Positive control, Cyclophosphamide monohydrate

M: Male F: Female NAD: No Abnormality Detected Bwt: Body weight

**Appendix 8.** Individual Animal Data - Chromosomal Aberrations and Mitotic Index

Ct: Chromatid CS: Chromosome MP: Metaphase plate BC: Blast cells

#: Metaphase plate with one or more aberrations considered as one metaphase plate with aberrationsMitotic index: No. of metaphase plates per 1000 blast cells

**Appendix 8.** cont. Individual Animal Data - Chromosomal Aberrations and Mitotic Index

Ct: Chromatid CS: Chromosome MP: Metaphase plate BC: Blast cells

#: Metaphase plate with one or more aberrations considered as one metaphase plate with aberrations Mitotic index: No. of metaphase plates per 1000 blast cells

**Appendix 8.** cont. Individual Animal Data - Chromosomal Aberrations and Mitotic

Ct: Chromatid CS: Chromosome MP: Metaphase plate BC: Blast cells

#: Metaphase plate with one or more aberrations considered as one metaphase plate with aberrations

Mitotic index: No. of metaphase plates per 1000 blast cells
